# Brownian orientational lath model (BOLD): A computational model relating the self-assembly in a fluid of lath like particles with its rheology and gelation

**DOI:** 10.1371/journal.pone.0191785

**Published:** 2018-02-07

**Authors:** Gabriel Villalobos

**Affiliations:** 1 Computational Biophysics, University of Twente, P.O. Box 217, 7500 AE, Enschede, The Netherlands; 2 Universidad de Bogotá Jorge Tadeo Lozano, Departamento de Ciencias Básicas, Carrera 4 Número 22 - 61. Módulo 6, oficina 501, 110311, Bogotá, Colombia; Stanford University, UNITED STATES

## Abstract

By means of a computational model, we study the relation between two complementary views of gelation, rheological tests against the characterization of a network of consecutive particles. The model we propose consists of slender, plane, colloidal sized particles, which we name laths, which self-assemble into long ordered aggregates of several particles; called whiskers in the literature. Within a whisker, the interaction potential is a minimum when: the planes of two consecutive laths are aligned, thus favoring their alignment; when the center of three consecutive laths lie in a straight line, thus favoring stacking; and when the center of two consecutive laths are located at a certain distance, which mimics excluded volume. A threshold value of the potential gives a condition for sticking free laths into whiskers, and for the breaking of whiskers. The simplicity of the model allows the simulation to reach large enough times, of the order of minutes, needed to simulate numerical rheology tests. We are able to characterize the whisker formation, as well as to simulate the gel transition, by means of an oscillatory shear numerical experiment. We conclude that according to the usual rheological definition a gel transition occurs at about 250*K*, even though there is no branching and less than 10% of whiskers are long enough as to percolate the system.

## Introduction

A gelation process corresponds to the formation of a network of chemical or physical bonds between the molecules or particles composing the liquid. It connects any chosen point of the non-fluid phase to another [1], and branching is essential [2]. In terms of rheology, the gel transition corresponds to a change between liquid-like to solid-like behavior, which can be seen as a cross in the behavior of the storage and loss modulus; for liquids *G*′′ ≫ *G*′, while for solids it is the reverse [2]. In this paper we study the gel transition of a model of slender, plane, colloidal sized particles that have an attractive interaction oriented mostly perpendicular to their areas. Such interaction is expected to cause long ordered aggregates, by means of alignment of the corresponding particles. With this model we address the relation between those two views of gelation.

To compare with experiments, we refer to the aggregates of Poly(3-hexylthiophene), molecules. For P3HT our model is just a rough estimate, since we do not take into account the flexibility of the molecule, and it is coarse grained into a single lath. However, our numerical simulations show a very good resemblance to the rheology of the system reported in the literature (as in [3–5]), showing that the highly coarse grained model suffices to represent most of the physics of this phenomenon.

Usually, Coarse Grained (CG) models represent a polymer molecule as a bead spring chain, with several molecules represented by a single blob [6, 7]; up to a complete polymer being coarse grained into a particle, for which the effect of the configuration of eliminated coordinates is included by a dynamic field [8]. We aim to a high degree of coarse graining, thus we retain just the basic elements of the system. The particles are *laths*, defined by two directions, one along the long axis and one perpendicular to it. The potential energy that generates the stacking depends on only one energetic parameter. It consists on the product of two functions, one that depends on the relative orientation among laths and one that represents the attraction of the laths in the direction perpendicular to the plane of the molecule and the excluded volume contribution. In combination with a Brownian dynamics method, this gives a fast and simple model which generates and allows studying the aggregation of the laths into whiskers. Our highly coarse grained model relates the structure of the network of particles with the macroscopic rheological measurements.

In order to characterize the gelation process of our model, we test the loss and storage modulus of the system while changing the temperature; providing a way to test the gelation process. We find the macroscopic evidence of gelation, in terms of a switch in the behavior of *G*′ and *G*′′, even though our present model does not allow the branching of whiskers.

Previous computational studies have used Langevin coarse grained and MD simulations to study self-assembly of P3HT into whiskers [9–11]; to study gel formation [12]; as have used an ab initio-based force field, for the molecular structure calculation [13]. In our view a minimal model for studying the rheology at the gel transition does not need to explicitly account for the forces between individual atoms or molecules. This can even make difficult to interpret the main causes of the gelation process. Our model, being simpler, is less expensive to implement in larger simulations and could be extended to study the form of the interface between P3HT and C60. It is similar to the study of gelation of patchy rod-like particles [14], but our system is not athermal and contrary to that reference, in our system the bonds between particles are allowed to break. To the best of our knowledge, no similar highly coarse grained simulations of stacking of particles capable of rheological measurements has been attempted, either in polymer or colloidal systems.

## Materials and methods

We name the model Brownian Orientational Lath Model, BOLD. The shape of the distance dependence of the potential is qualitatively similar to that found in the literature for thiophene [15, 16], and the location of the minimum of the potential corresponds to the distance between planes in the crystals of P3HT [17, 18]. The rules for sticking and unsticking are meant to model the whisker evolution.

### Self-assembly and whisker formation model

#### Configurations and interactions

**Fig 1:**
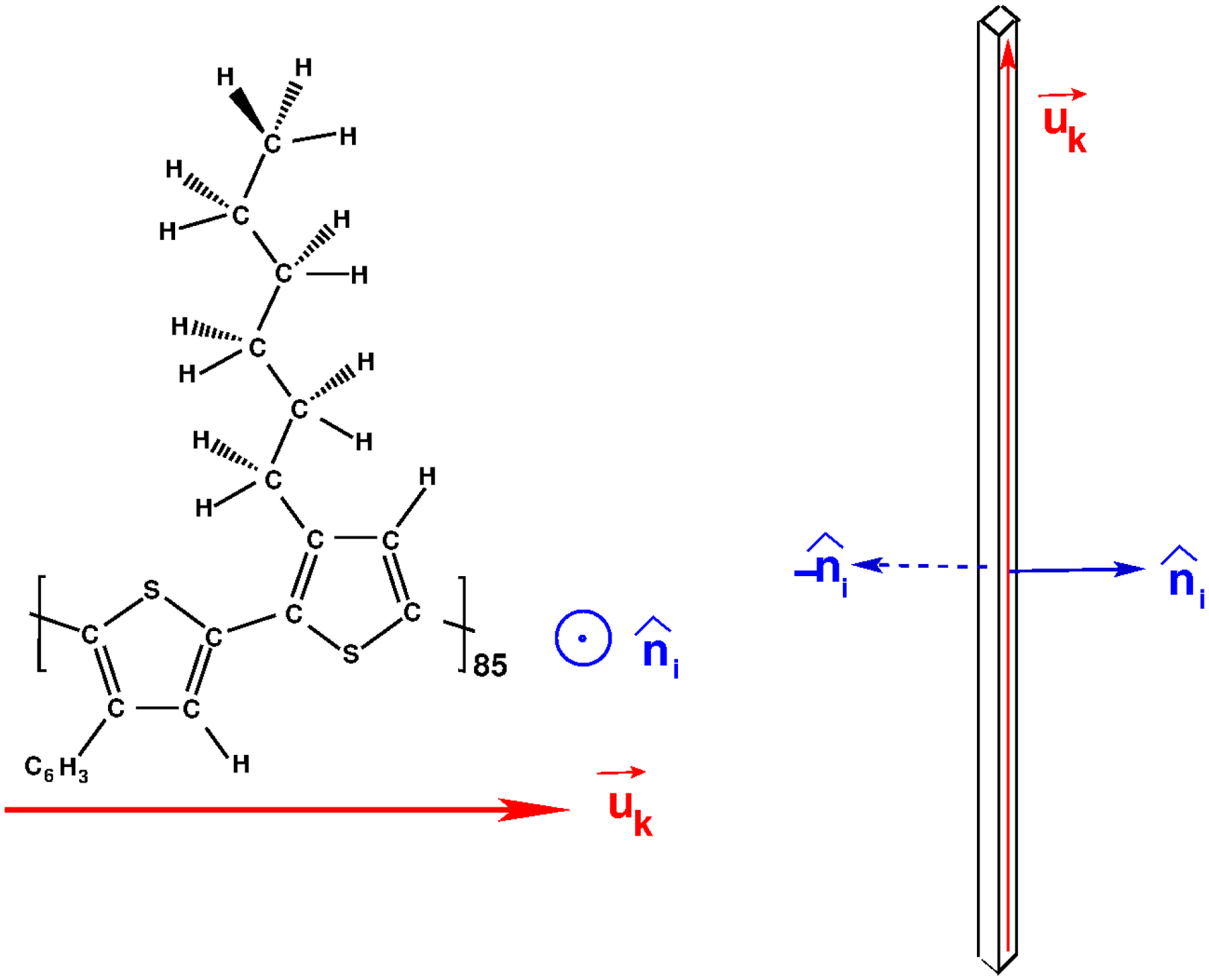
Molecular structure of P3HT and lath aspect ratio. *(Left)* Molecular structure of two thiophene rings of P3HT [13]. A single lath will have 85 such units along the backbone. *(Right)* Three dimensional sketch of a lath, with two $\hat{n}$ vectors, the sketch represents the actual aspect ratio of length (along backbone) to width. The direction ${\hat{u}}_{k}$, red, is defined along the backbone of the molecule; the normal $\hat{n}$, blue, is defined perpendicular to the plane of the molecule. This is further explained in Fig 2.

We investigate the gel transition of strongly interacting lath-like objects of colloidal sizes. Although we relate the results of our model to the experimental system of *π*-stacking polythiophenes in solution, the model itself is as generic as possible, and the results of the computational model can be interpreted in its own merit. The basic object of our simulations is a lath, depicted in Fig 1. We describe its position by the three Cartesian coordinates **r** = {*x*, *y*, *z*} of its center of mass, and its orientation in space by and the two perpendicular unit vectors $\hat{u}$ and $\hat{n}$. Subscripts indicate the particular lath under consideration. The vector $\hat{u}$ specifies the direction of the long axis of the lath while $\hat{n}$ is chosen to be perpendicular to the plane spanned by the two longest edges of the lath. In all that follows it is assumed that the shortest of the three edges is much shorter than the others, as is the case in P3HT.

**Fig 2:**
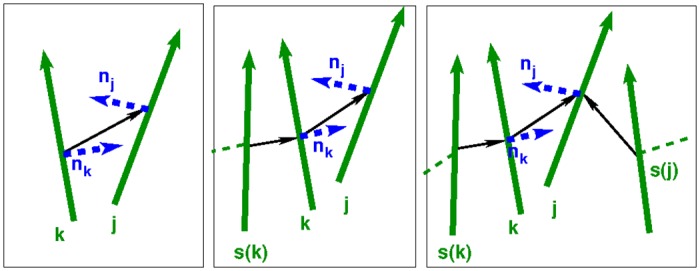
Sketch of the vectors used in the potential. In bold continuous (green), the orientational vectors ${\hat{u}}_{k}$; in thin continuous (black), the connectors; in thick dashed (blue), the normal vector **n**_*k*_; finally in thin dashed (green), possible connectors to other laths. The present model is 3D but to simplify the sketch, vectors are represented in 2D. *s*(*k*) is stuck to *k*, and *k* and *j* may or may not be stuck together. (*left*) Neither *k* nor *j* is stuck to any other lath (say *l*), then we pick **n**_*k*_ and **n**_*j*_ using the components of the connector that are perpendicular to the respective orientation vectors. (*center*) *k* is stuck to *s*(*k*) ≠ *j*, *j* does not have another stuck particle; the normal in *k* is given by its interaction with its stuck particle *s*(*k*). (*right*) Both *k* and *j* are stuck to some other laths.

We propose to describe the potential of a given configuration as

$$\begin{array}{c} \Phi_{S}=\epsilon \sum_{j,k}V_{d}\left(r_{kj}\right)V_{o}\left({\hat{u}}_{k},{\hat{u}}_{j}\right)V_{cn}\left({\hat{n}}_{k}\cdot r_{kj}\right)V_{cn}\left({\hat{n}}_{j}\cdot r_{kj}\right) \end{array}$$ (1)

where **r**_*kj*_ = **r**_*k*_ − **r**_*j*_, is connector between two laths, and *r*_*kj*_ is its length, as seen in Fig 2. The first factor in each term describes the dependence of the interaction energy on the distance between the centers of mass of the two laths under consideration, and the three other factors describe the dependence on their relative orientations. Explicit expressions are:

$$\begin{array}{ccc} V_{d}\left(r_{kj}\right) & = & \frac{1}{2}\left(\frac{\tanh(a\left(r_{kj}-\frac{\sigma }{2}\right))}{\tanh(\frac{a\sigma }{2})}-1+\frac{A_{excl}}{{\left(r-r_{excl}\right)}^{4}}\right)\\ V_{o}\left({\hat{u}}_{k},{\hat{u}}_{j}\right) & = & {\left({\hat{u}}_{k}\cdot {\hat{u}}_{j}\right)}^{2}\\ V_{cn}\left({\hat{n}}_{k},{\hat{r}}_{kj}\right) & = & \left\{ \begin{array}{cc} {\left({\hat{n}}_{k}\cdot {\hat{r}}_{kj}\right)}^{2} & \text{if}\;{\hat{n}}_{k}\cdot {\hat{r}}_{kj}>0\\ 0 & \;\text{else} \end{array}\right. \end{array}$$

**Fig 3:**
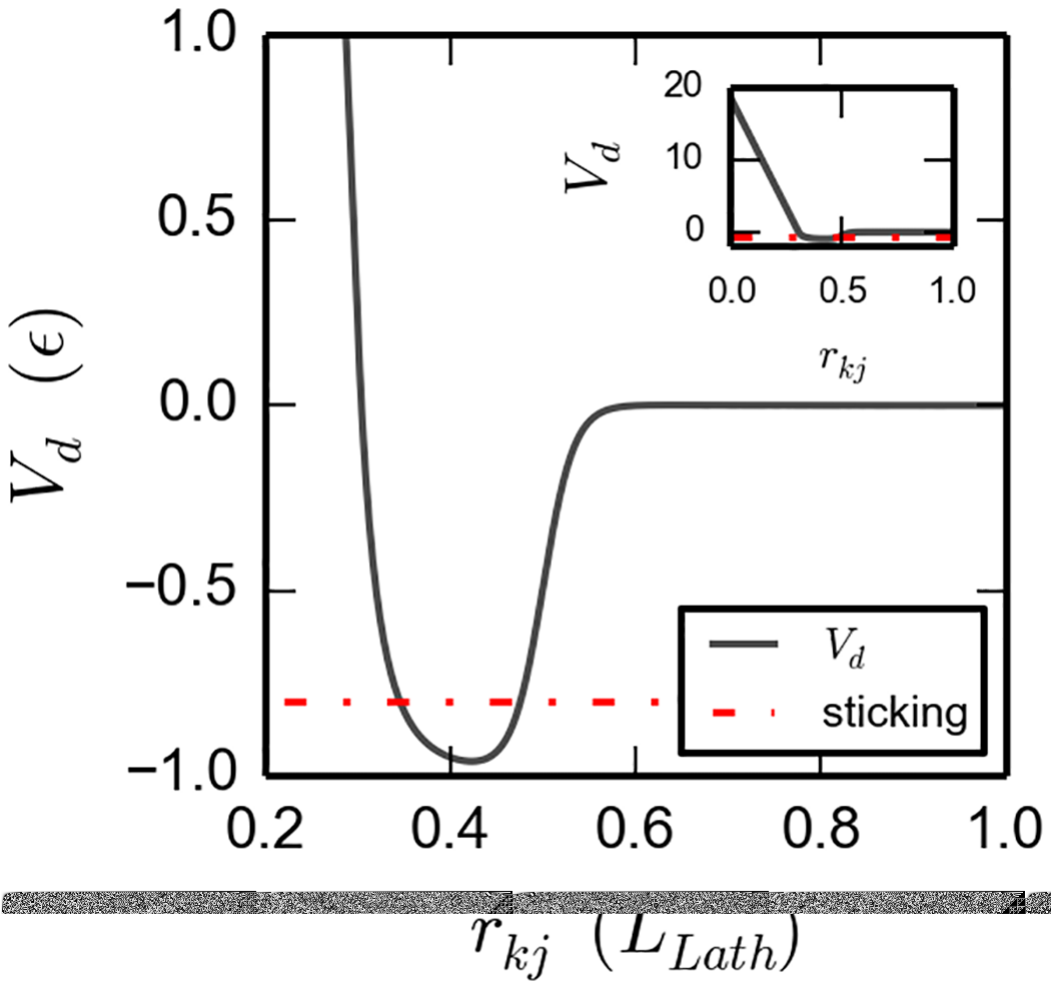
Dependence of the potential with the distance, *V*_*d*_(*r*_*k*, *j*_). It is a narrow function whose center is close to 0.4 *L*_*lath*_. Below a cutoff, *r*_*cutoff*_ = 0.3 *L*_*lath*_, it is replaced with a linear extension. The threshold for sticking and unsticking lays close to the bottom of the well, 0.2*ϵ* above the absolute bottom, (dash-dotted red line). Inset shows the same potential, with a different scale.

The second factor, $V_{o}\left({\hat{u}}_{k},{\hat{u}}_{j}\right)$, favors a parallel orientation for the long axis of the two laths. While the last two factors, $V_{cn}\left({\hat{n}}_{k}\cdot r_{kj}\right)$ and $V_{cn}\left({\hat{n}}_{j}\cdot r_{kj}\right)$, minimize the energy when the two vectors ${\hat{n}}_{k}$ and ${\hat{n}}_{j}$ are both parallel to the connector **r**_*kj*_, thereby favoring the two faces of the laths to be parallel. Our reason for distinguishing between positive and negative values of ${\hat{n}}_{j}\cdot r_{kj}$ will be explained below. The specific functional form, quadratic in ${\hat{n}}_{j}\cdot r_{kj}$, is of no special relevance, only the fact that is an even function. To the best of our knowledge, the specific functional form of *V*_*d*_(*r*_*kj*_) has not been determined from a calculation based on first principles. Therefore, we have decided to model a wheel potential with a defined minimum, in the first term; as well as an excluded volume, in the second term (*V*_*d*_(*r*_*kj*_) is plotted in Fig 3. The minimum is seen to be at a distance between two laths of about 0.4 ∗ *L*_*lath*_; implying that the thickness of the lath plus the face-to-face distance is about equal to this value. In the simulations, the potential is linearized below a 0.3 ∗ *L*_*lath*_ to allow for acceptable time steps, as is the value of *σ*/2. The values of the various parameters in *V*_*d*_(*r*_*kj*_) are given in Table 1, and they are further discussed below.

**Table 1:** Parameters used in the model.

Parameter	Symbol	Value
Strength of the alignment potential.	*ϵ*	4.04 × 10^−19^ *J* = 100*k*_*B*_ × *T*_0_
Length of the laths, unit of distance	*L*_*lath*_	68.44 *nm* ≈ 177 thiophene rings
Length of the edge of the cubic simulation box	*L*_*box*_	8 × *L*_*lath*_ = 0.5442*μm*
Laths in the simulation box	*N*_*laths*,*total*_	512
Lath density		10.8 *g*/*l*.
Lath number density		3.1193 × 10^21^ particles/*m*^3^.
Inflection point of *V*_*d*_	*σ*/2	34.22 *nm*
Cutoff for the linear extension of *V*_*d*_	*r*_*cutoff*_	0.3*L*_*lath*_ ≈ 20.53*nm*
Threshold for sticking/unsticking of laths.	*V*_*thr*._	−0.8*ϵ*
Slope (sharpness) of potential *V*_*d*_	*a*	20*m*^−1^
Excluded volume distance constant	*r*_*excluded*_	0.22*L*_*lath*_ ≈ 15.05*nm*
Excluded volume amplitude	*A*_*excluded*_	0.0001
Time-step	Δ_*t*_	1 × 10^−5^ *s*
Initial Temperature	*T*_0_	293 *K*
Solvent friction (translational)	*ξ*_0_	8.66 × 10^−7^ *kg*/*s*
Solvent friction (rotational)	$\xi_{0t}=\frac{\xi_{0}}{9}$	9.62 × 10^−8^ *kg*/*s*
unit of time	$L_{Lath}^{2}/D_{0}$	1.0762 *s*
unit of velocity	*D*_0_/*L*_*Lath*_	6.3592 × 10^−8^ *m*/*s*
unit of pressure	$k_{B}T_{0}/L_{Lath}^{3}$	11.7571 *Pa*
Diffusion coefficient	$D_{0}=\frac{k_{B}T_{0}}{\xi_{0}}$	4.3522 × 10^−15^ $\frac{m^{2}}{s}$
Amplitude of oscillation pressure	$\frac{1}{2}k_{B}T_{0}/L_{Lath}^{3}$	5.875 *Pa*

Within a whisker, a lath is strongly bound to one; or, more commonly, to two other laths. In order to complete the description of the potential energy, we must define when this binding within two laths happens. This will be the case when their interaction energy is less than a threshold energy *V*_*thr*_ for which we choose *V*_*thr*_ = −0.8*ϵ*. Then, two laths which are strongly bound to each other, while none of the two laths is strongly bound to another lath, interact like a pair of free laths.

Take notice that the design of the potential does not allow branching to happen, which allows for us to study gelation in a branch-free system.

#### Propagator and refinement of potential

There is no qualitative difference between the dynamics of ${\hat{u}}_{k}$ and that of ${\hat{n}}_{k}$, a simulation shall treat both with the same methods and precision. However, ${\hat{n}}_{k}$ is by definition perpendicular to ${\hat{u}}_{k}$, so that ${\hat{u}}_{k}$ has three degrees of freedom and ${\hat{n}}_{k}$ has one less. In the case of lath-like particles, rotations around the long axes will be much faster than re orientations of ${\hat{u}}_{k}$ or displacements of the laths as a whole; in other words, the dynamics of ${\hat{n}}_{k}$ will be much faster than that of ${\hat{u}}_{k}$. Therefore, we assume that during one time step the lath has fully explored all possible orientations around its long axis and has settled into the corresponding energetic minimum. This means that, during the simulation, the vector ${\hat{n}}_{k}$ will be determined by the configuration described by $\left\{r_{k},{\hat{u}}_{k}\right\}$ and its recent history (see below). We are then left with two equations of motion for each particle:

$$\begin{array}{ccc} dr_{k} & = & -\frac{1}{\xi_{0}}\nabla_{k}\Phi_{C}dt+\Theta_{k}^{t}\sqrt{\frac{2k_{B}Tdt}{\xi_{0}}} \end{array}$$ (2)

$$\begin{array}{ccc} d{\hat{u}}_{k} & = & \frac{L_{lath}^{2}}{9\xi_{0}}T\times {\hat{u}}_{k}dt+\Theta_{k}^{r}\frac{L_{lath}}{3}\sqrt{\frac{2k_{B}Tdt}{\xi_{0}}} \end{array}$$ (3)

Here $\Theta_{k}^{t}$ is a three dimensional random vector with components which have zero mean and unit variance, uncorrelated among each other. Similarly, $\Theta_{k}^{r}$ is a two dimensional random vector with uncorrelated components having zero mean and unit variance. The two random rotations are applied around two perpendicular axes orthogonal to ${\hat{u}}_{k}$. After each time step the length of the vector ${\hat{u}}_{k}$ is brought back to unity by a shortening along the original orientation [19, 20]. *ξ*_0_ is the average translational friction of the lath; we have chosen the rotational friction to be related to the translational friction as for infinitely long rods [20, 21], and friction is assumed to be isotropic.

Let us describe how we update ${\hat{n}}_{k}$. First, consider two laths *k* and *j*, neither of whom is interacting with any other lath. We choose $n_{k}=r_{kj}-\left(r_{kj}\cdot {\hat{u}}_{k}\right){\hat{u}}_{k}$, and similarly for ${\hat{n}}_{j}$, since these are the orientations assumed to correspond to the minimum interaction between the two laths for the given configuration $\left\{r_{kj},{\hat{u}}_{k},{\hat{u}}_{j}\right\}$. The unit vector ${\hat{n}}_{k}$ is $n_{k}/\sqrt{n_{k}\cdot n_{k}}$. This choice will drive the two laths to become parallel and have their centers of mass connector **r**_*kj*_ perpendicular to both ${\hat{u}}_{k}$ and ${\hat{u}}_{j}$. Substituting these expressions for ${\hat{n}}_{k}$ and ${\hat{n}}_{j}$ into the potential above leaves us with a simple pair potential as commonly used in simulations of hard convex bodies.

The situation is different when a free lath *j* interacts with a lath *k*, which is strongly interacting with another lath *s*(*k*) ≠ *j*. In this case lath *j* may still freely reorient along its long axis, but lath *k* is restricted to do so by its strong interaction with *s*(*k*) ≠ *j*. We therefore choose ${\hat{n}}_{j}$ as above, but set ${\hat{n}}_{k}$ equal to the normalized $n_{k}=r_{s(k),k}-\left(r_{s(k),k}\cdot {\hat{u}}_{k}\right){\hat{u}}_{k}$. The *s*(*k*) is strongly bound to lath *k*. We are now in the situation where we need to distinguish between two cases in the definition of *V*_*cn*_. Only when lath *j* is situated on the positive side of lath *k* it may bind to lath *k*; on the negative side lath *k* is already bound to lath *s*(*k*) and cannot bind to the newly arriving lath *j* anymore. This treatment of the re-orientations of the laths around their long axes turns the potential in this case into a three body potential. Moreover, the potential becomes history dependent. This is similar to coarse grain simulations of linear polymers [22], where the entangling of polymers is not fully determined by the configuration of the beads, but depends on how the beads came to that particular configuration.

A third situation occurs when two laths *k* and *j* approach each other, but are already bound to *s*(*k*) ≠ *j* and *s*(*j*) ≠ *k* respectively. In this case we have $n_{k}=r_{s(k),k}-\left(r_{s(k),k}\cdot {\hat{u}}_{k}\right){\hat{u}}_{k}$; and similarly for **n**_*j*_. Only when lath *j* is on the positive side of lath *k*, determined by lath *s*(*k*), and lath *k* is on the positive side of lath *j*, determined by *s*(*j*), will the interaction be non-zero. This treatment turns the interaction into a four-body interaction, (see Fig 2).

The explicit expressions for forces and torques appear in S1 and S2 Files.

#### Sticking-unsticking dynamics

A lath is defined to remain *free* if the energy of interaction with all its neighbors is above a minimum threshold (small in absolute value or positive). On the contrary a lath within a whisker is *stuck* to its two closest neighbors and does not interact with the others. A lath at the end of the whisker is stuck to one, and interacts with the free laths. For any given configuration of the system the interaction energies for pairs of laths is history dependent, since two laths may be quite close and their **u** vectors highly aligned, but they may not interact strongly, being part of different whiskers. Incidentally, regarding the simulation method, notice that dependence of the state of the system with the past makes it more intricate to use a dynamic Monte Carlo than a Brownian Dynamics, since the geometric arrangement does not univocally define the whiskers.

At the beginning of the simulation the interaction energy between pairs of laths is calculated. For each lath energies are ordered from lowest to highest; and then they are also ordered from lowest to highest among every lath. We then follow this ordered list to assign the neighbors. For each particle of this list, index *k*, we check whether it is stuck to other particles. If it has none or one stuck particles, then it can stick, therefore we check whether the lowest energy of interaction is below the threshold. If it is, then the particle *k* gets stuck to the corresponding one, say *j*, and both get marked as stuck. During this step of the interaction *k* and *j* are marked so they are not checked anymore for sticking in this step. We continue going through the laths in the list, until all of them have been checked. Afterward, we check for unsticking. If the interaction energy of a given pair of laths no longer is below the threshold, then they are no longer stuck. With this process we ensure that whiskers form and break according to the current state of the system and the interaction energies between free and stuck laths.

### Parameter settings and simulation conditions

The space of the simulation is a cubic box whose edge measures 8 times the length of a lath, or 0.5442 *μm* (see Table 1). Most of the simulations are run with 512 laths, which implies a density of 1 $\text{lath}/L_{lath}^{3}$. In SI, this corresponds to a density of 10.8 *g*/*l*. We use periodic boundary conditions, [23] and in those simulations in which shear flow is applied with a velocity gradient $\dot{\gamma }$, we implement Lees-Edwards sliding boundary conditions [23]. Each time-step corresponds to 10^−5^ *s*. The total simulation time of each set of simulations is included in the caption of each figure.

A set of nondimensiolanized units are given by the average translational diffusion coefficient of the laths *D*_0_ = *k*_*B*_ *T*_0_/*ξ*_0_, the thermal energy *k*_*B*_ *T*_0_ and the length of a lath *L*_*Lath*_. With this choice, the unit of time is $L_{Lath}^{2}/D_{0}$, the unit of velocity is *D*_0_/*L*_*Lath*_, and the unit of pressure is $k_{B}T_{0}/L_{Lath}^{3}$. Numerical values are given in Table 1. However, SI units are used in the description of the results of this paper. Table 1 contains all model parameters used in this study. Parameter settings are meant to be reasonably close to those that apply to P3HT solutions.

Since it is a general model, we are free to choose the length of the lath, and the strength of the potential. However, to compare with an experimental system, we have chosen to match the length to commercially available P3HT. A molecular weight of 29200 *g*/*mol*, gives about 176 thiophene rings in each molecule. Since the aspect ratio of length to width of such molecule is 68.44/1.2 ≈ 57, the shape is closer to a rod than an ellipsoid; therefore we use the friction coefficient for that geometry (Figure 2 of [24]).

We are unaware of any experimental studies of inter molecular interactions in thiophene. Therefore, we rely on theoretical methods, as MP2, MP4, CCSD and CCSD(T) [25]. For the value of the thiophene interaction energy Rodríguez et. al. report −0.34 *kcal*/*mol* [26]. Since the energy of interaction of van der Waals systems do not scale linearly, this gives a lower bond on the value of the interaction (disregarding n-body interactions, which Rodríguez et. al. claim to be negligible.) We pick the lath-lath interaction energy of *ϵ* = 100*k*_*B*_ *T*_0_ = 4.04 × 10^−19^ *J* = 2.5215*eV* = 243.2877*kJ*/*mol*, or −0.33 *kcal*/*mol* per thiophene pair, emphasizing that the important characteristics of the model are that bonding can only occur in one-dimensional structures and that the bonding is very strong, *i*.*e*. *ϵ* = 100*k*_*B*_ *T*_0_. Further discussion of these values is included in the S3 File.

**Fig 4:**
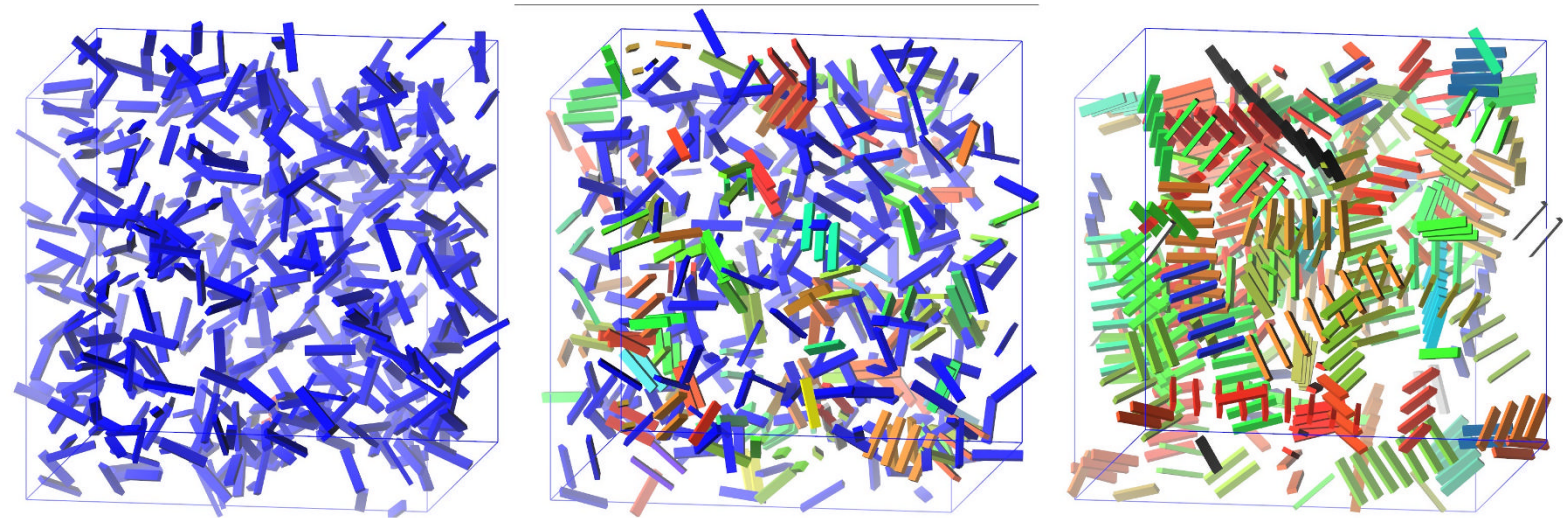
Snapshots of one simulation. The aspect ratio is not at scale, for easier visualization (a realistic aspect ratio is depicted in Fig 1). For this simulation: *T* ≈ 0.1*T*_0_, other parameters in Table 1. *(Left)*: Initial state of the simulation. Different whiskers have different colors. Blue laths are free. *(Middle)*: *t* = 0.5 *s*, starting to create some whiskers. *(Right)*: At *t* = 200 *s*, several long whiskers.

Results from a typical run at *T* = 0.1*T*_0_ are shown in Fig 4. The left panel box shows the beginning of the run. All laths are colored blue, none of them is strongly bound to any other. In the middle panel the same system is shown at *t* = 50000, or 0.5 *seconds*. Several laths are connected through a sequence of consecutive strong bonds, they are part of the same whisker, and are given the same color. In the right panel, the same box is shown at *t* = 20000000, (200 *seconds*). The system has reached dynamic equilibrium, the average quantities have stabilized; and several long whiskers are observed.

### Dynamical properties

Following Oliveira et. al., we obtain the linear rheology of the fluid by computing the auto correlation of the shear stress [27]. The shear stress tensor reads:

$$\begin{array}{c} S_{xy}\left(t\right)=-\frac{1}{V}\sum_{i,j}\left(r_{i,x}-r_{j,x}\right)F_{ij,y} \end{array}$$ (4)

where *F*_*ij*,*y*_ is the *y*-component of the force on particle *i* due to the interaction with particle *j*. The auto correlation of the shear stress gives the shear stress relaxation modulus:

$$\begin{array}{c} G\left(t\right)=\frac{V}{k_{B}T}<S_{xy}\left(t\right)S_{xy}\left(0\right)> \end{array}$$ (5)

It is possible to take the real and imaginary parts of the Fourier transform of *G*(*t*) to obtain the storage modulus *G*′ and the loss modulus *G*′′. In this paper we used a different approach for these particular rheological measurements. In order to simulate the oscillatory shear experiment, the system forced applying an oscillatory dependent strain:

$$\begin{array}{c} \gamma =\gamma_{0}\sin\left(\omega t\right) \end{array}$$ (6)

with *ω* = 1 Hz, and *γ*_0_ = 0.5*L*_*lath*_. The resulting *σ*_*xy*_ is given by:

$$\begin{array}{c} \sigma_{xy}=\int_{-\infty }^{t}dt^{\prime }G\left(t-t^{\prime }\right)\gamma \left(t^{\prime }\right) \end{array}$$ (7)

After taking the integral and using the standard definition of *G*′ and *G*′′, one obtains:

$$\begin{array}{c} \frac{\sigma_{xy}}{\gamma_{0}}=G^{\prime \prime }\cos\left(\omega t\right)+G^{\prime }\sin\left(\omega t\right) \end{array}$$ (8)

We apply nonlinear fitting to obtain the value of *G*′ and *G*′′ from the value of *σ*_*xy*_/*γ*_0_ that is measured in the simulation.

## Results

First we describe the approach to equilibrium of simulations boxes which were initially either fully disordered or fully ordered. This gives information about typical time scales in the system. We also analyze the structure of final equilibrium boxes. In a second subsection we describe the rheological properties of our systems, with special attention being given to the gel transition.

Since our goal is to study the relationship between the usual rheological definition of a gel transition and the structure of the network of particles properties, we run simulations in a wide range of temperatures; even though it is clear that for the extreme values of temperature the similarity with P3HT is no longer valid. The glass transition for pure P3HT is 285.25 *K*, [28], and has a melting temperature of 505.86 *K* [29].

These results have been made publicly available online [30].

### Time evolution of formation of whiskers and equilibrium distributions of lengths

As we have seen, the model parameters chosen in this study lead to the formation of whiskers, with simulations which started from completely disordered boxes. In this section we analyze how this structure evolves with time. To fully characterize the structure of the whiskers we studied several characteristics: length as a function of time, number of laths per whisker; number of whiskers formed in the simulation box, as a function of time; total material in whiskers as function of time, and histogram of distribution of whisker sizes.

**Fig 5:**
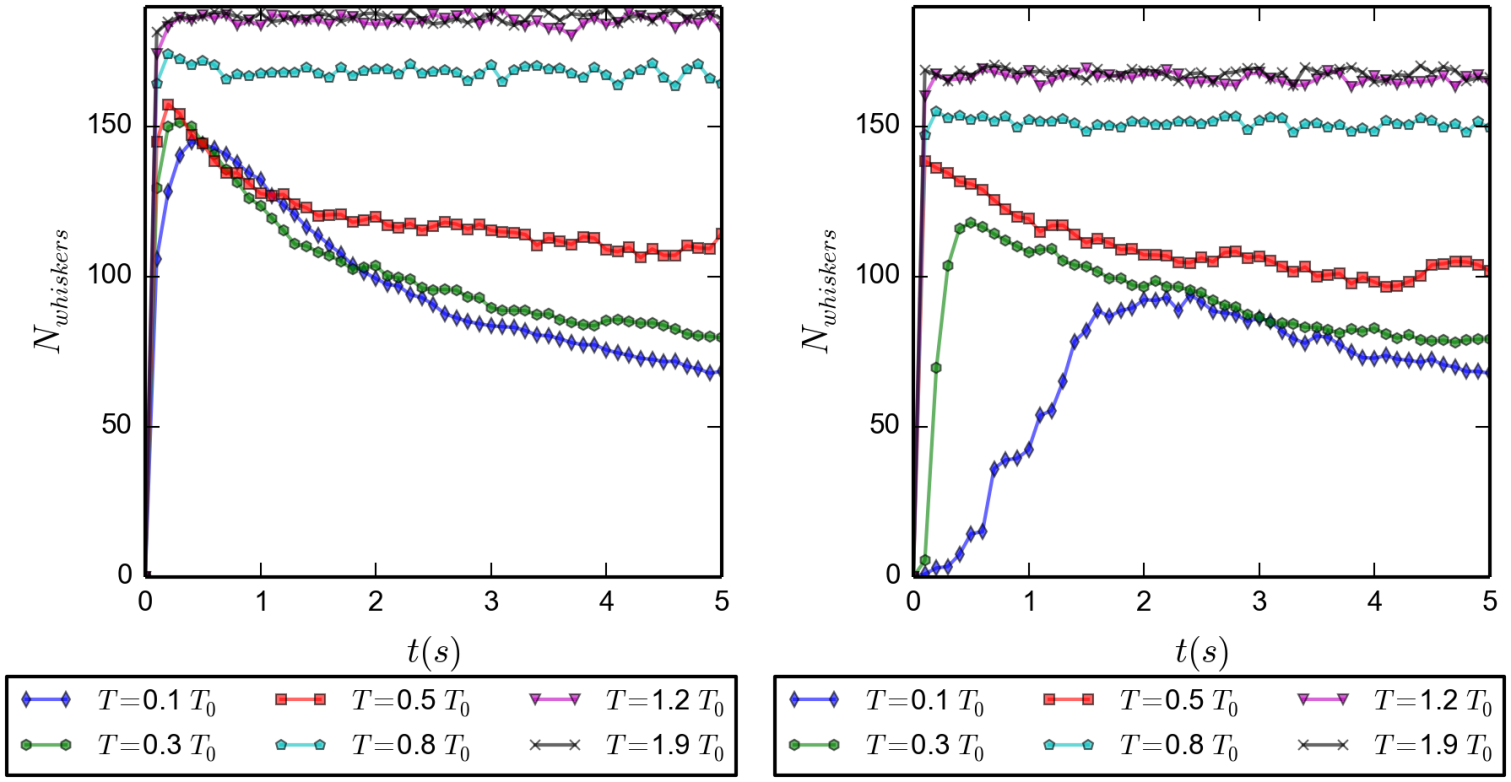
Number of whiskers as function of time. For 6 different temperatures; first 5 *s*; average over 20 simulations. *(Left)* initially disordered configuration, the number of whiskers reaches a higher plateau for higher temperatures. *(Right)* initially ordered configuration. The only discernible difference between them after a few seconds is a lower plateau for the high temperature average number of whiskers in the case of initially ordered simulation.

In Fig 5 we present, for various temperatures, the time evolution of the number of whiskers. The left panel corresponds to those that started with randomly distributed laths, while the right hand side panel presents boxes starting from fully order, *i.e.* those in which all laths were collected in one long whisker. Only the first five seconds are shown. The initially ordered simulations serve as a test of the consistency of the model.

For temperatures substantially larger than *T*_0_ (*T*_0_ = 293 *K*, Table 1), large numbers of whiskers (necessarily small) are observed after the first one tenth of a second. In boxes starting from disorder these are obtained by aggregation of laths, while in those starting from the ordered structure whiskers are obtained by disintegration of the initially available very long one. After the first 1/10 *s* have passed, hardly any changes occur in the number of whiskers. Notice that the various plateau values are not equal for corresponding temperatures in the left and right hand side panels. This is due to the fact that with our code the identification of whiskers is history dependent.

For temperatures of the order of *T*_0_ or less, in boxes starting from disorder, again numerous whiskers are formed within the first few tenths of a second. From then on the number of whiskers start to gradually decrease with increasing time. This is due to the fact that initially only very small whiskers are formed, which then start to merge into longer whiskers. Eventually, equilibrium distributions of lengths occur, depending on the temperature. For these temperatures, boxes starting from ordered configurations behave only slightly differently than those starting from disorder. Again, as in the high temperature case, long initial whiskers start to disintegrate, but this time increasingly slower with decreasing temperatures. This difference in speed of disintegration is not very important, as we do care most of the equilibrium distribution of initially disordered systems. There are still overshoots in the numbers of whiskers after which the distributions of whiskers begin to rearrange in order to approach the appropriate equilibrium distributions.

In Fig 5, as well as the following ones, we include a high temperature *T* = 550 *K* ≈1.9*T*_0_ The goal of making simulations at this temperature is to show the trend that would have increasing the temperature keeping all the other interactions unchanged, in other words, the asymptotic behavior of the system for very high temperatures.

**Fig 6:**
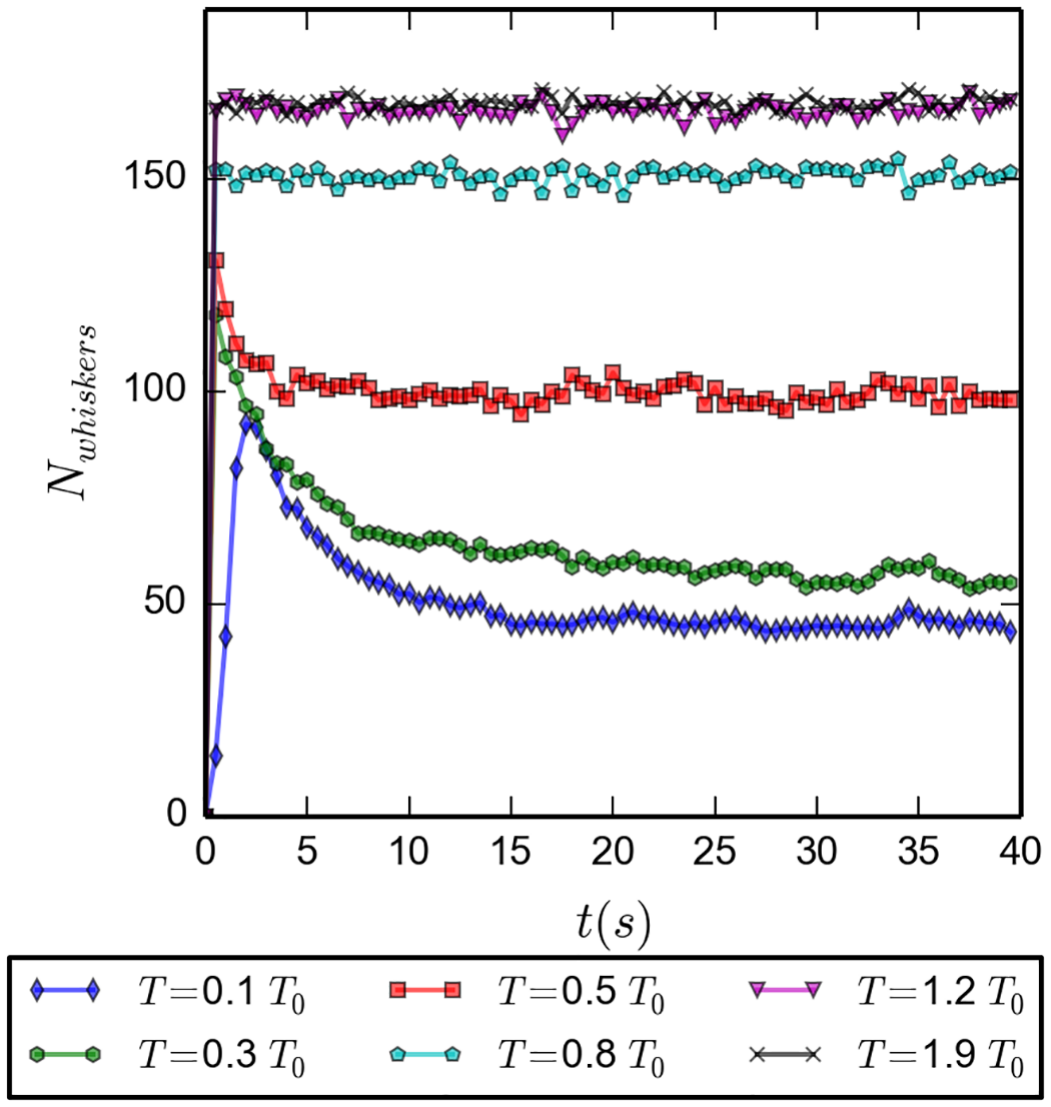
Number of whiskers as function of time for different temperatures; average over 20 simulations; initially ordered configuration; ran through 40 *s*. After the initial sharp growth in number of whiskers has happened, there is a decay to a plateau value that depends on the temperature.

In Fig 6 we present the time evolution of the number of whiskers for the same temperatures as above, but this time for a much longer time span of 40 *s*. Since there is no difference after the first five seconds between the simulations starting from disorder and those starting from order, except for the very high temperatures, we restrict the data here to those of boxes starting from order. The conclusion from theses runs is that it is safe to assume that equilibrium distributions have developed only after about twenty to twenty five seconds, depending on temperature.

**Fig 7:**
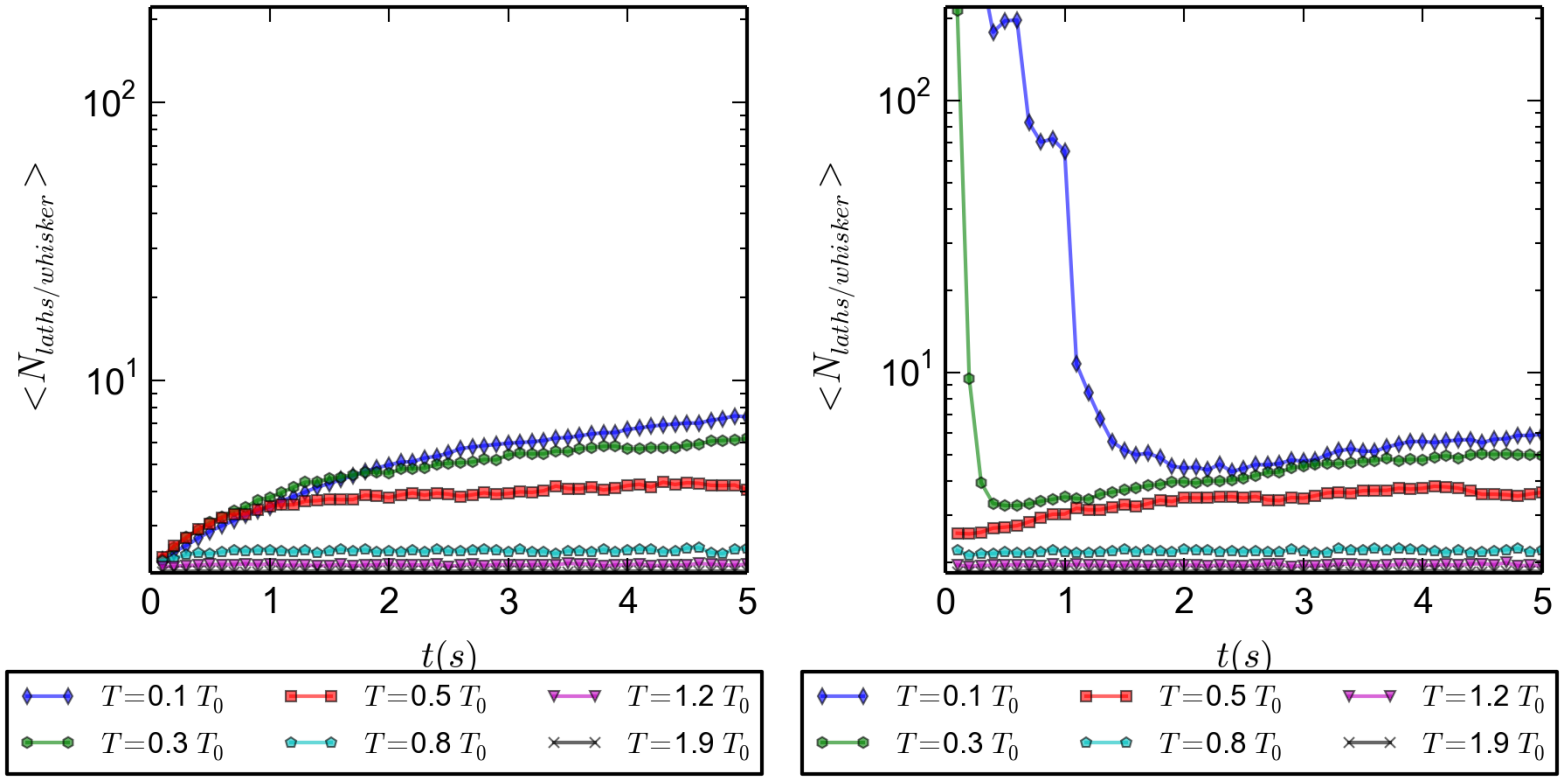
Average number of laths per whisker as function of temperature, for 20 simulations. In both panels the vertical axis are set in logarithmic scale, and the time runs for 5 *s*. *(Left)* initially disordered configuration, *(Right)* initially ordered configuration.

**Fig 8:**
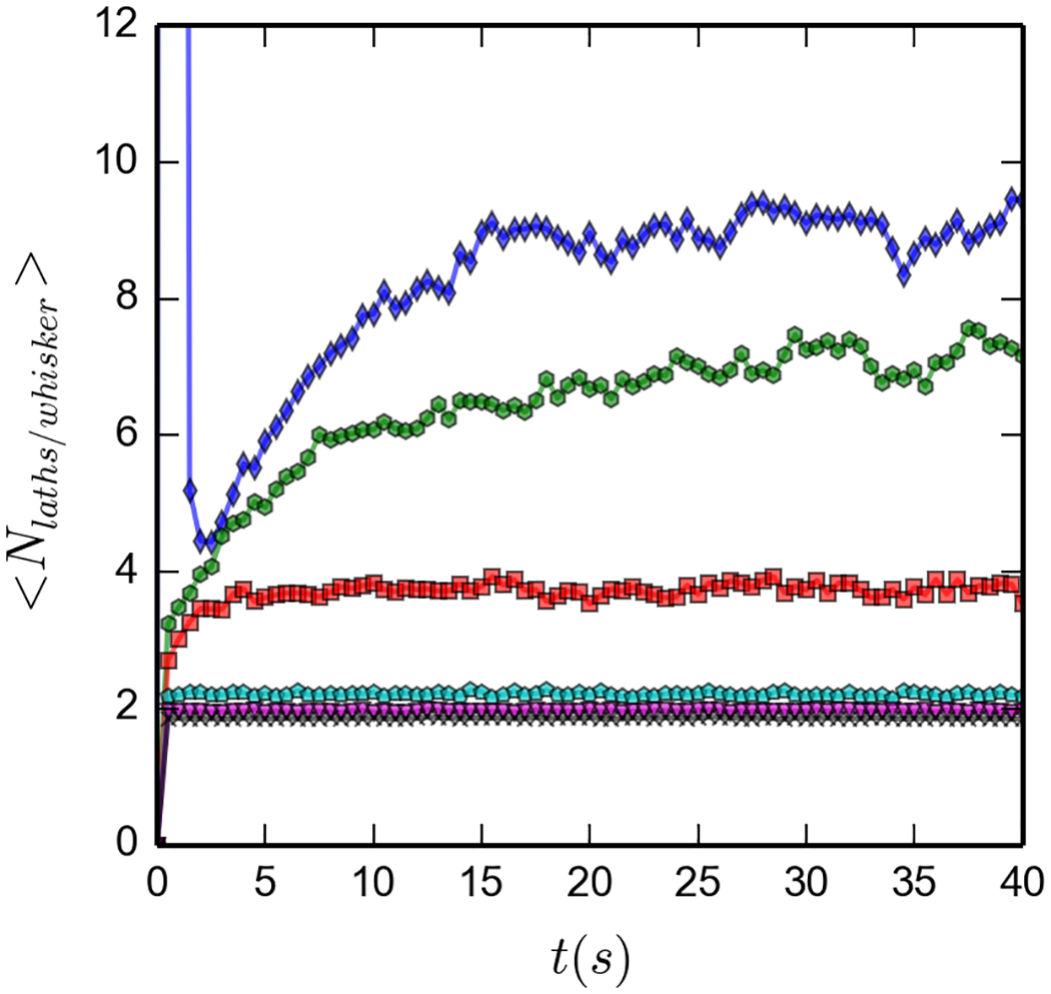
Average number of laths per whisker as function of temperature; during 40 *s*; initially ordered configuration; averaged over 20 simulations. Due to a different vertical scale, not all the data points of Fig 7 are seen here. Symbols and colors are the same as in Fig 7

Fig 7 shows the transient behavior, initial 5 seconds, of the average number of laths per whisker, <*N*_*laths*/*whisker*_>, for both initially disordered and ordered configurations. Vertical axes are on log scales to more easily span a broad range of values. Both plots show the expected behavior: a steady increase of the number of laths per whisker, for the initially disordered configurations; a breaking up of the initially available whisker spanning the whole box, followed by a steady increase of the number of laths per whisker, for the initially ordered simulations. The lower the temperature, the slower is the time evolution of the towards the final plateau values. Fig 8 shows the long time behavior, up to 40 *s*, for the initially ordered simulations. Up to twenty five seconds are needed before the systems reach equilibrium. High temperature systems reach only average values of two laths per whisker, the minimum value to form a whisker; as the temperature decreases the plateau values grow to values of about ten for the lowest temperature. As temperature increases, longer whiskers are broken into shorter ones, reducing the number of laths per whisker.

**Fig 9:**
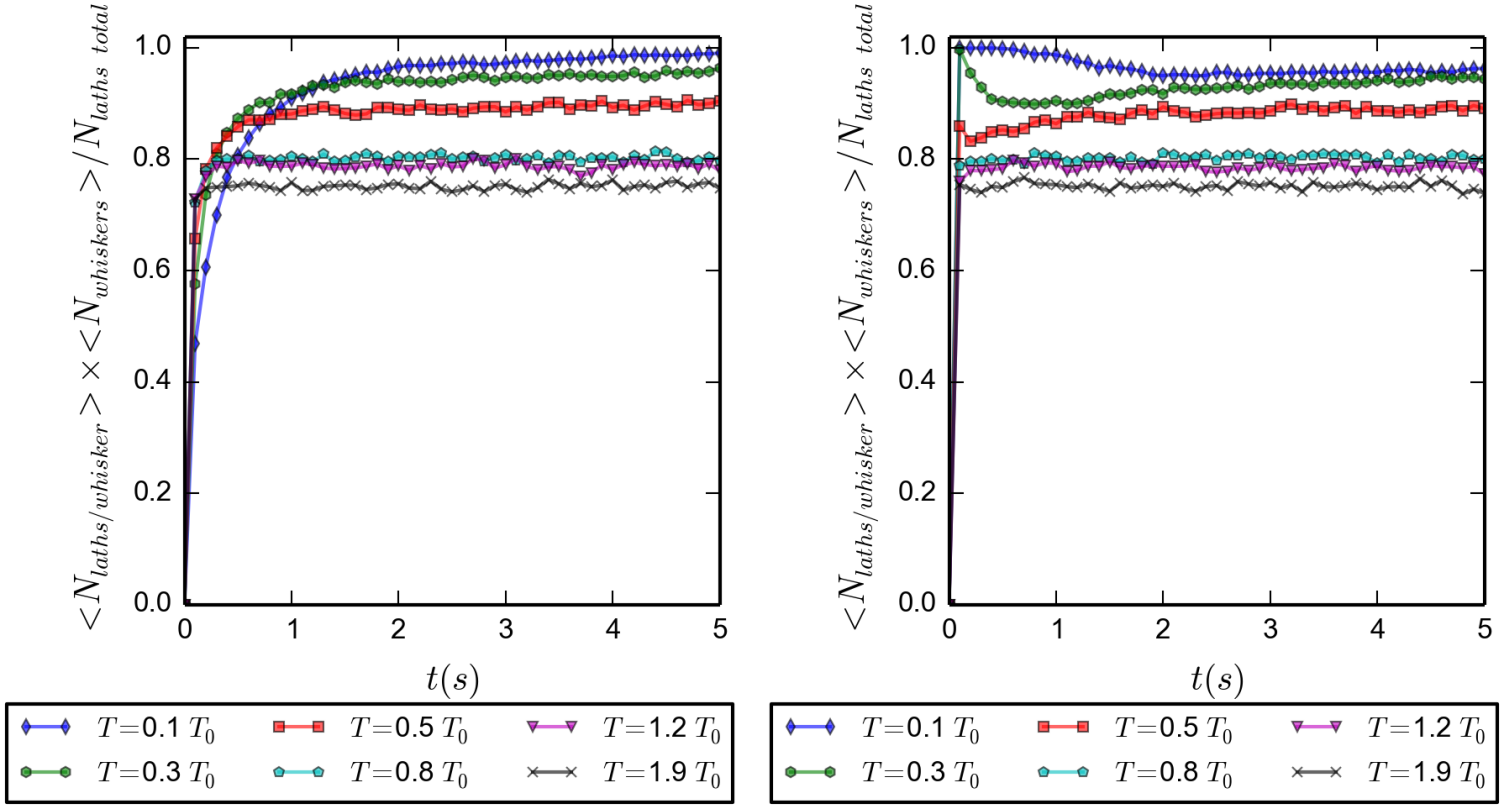
Fraction of the total material in whiskers as the product of the average number of whiskers and the average laths per whisker, divided by the total number of particles, for different temperatures (in units of *T*_0_); as a function of time and for the first 5 *s* of the evolution. *(Left)* Initially disordered configuration. *(Right)* Initially ordered configuration.

Have a look at the time evolution of the ratio of the total number of laths taking part in whiskers over the total number of whiskers, shown in Fig 9. Left panel results refer to systems starting from disorder, while the right one to those starting from order. In systems starting from disorder the total material in whiskers is monotonically increasing, while in those starting from order the total material in whiskers is almost constant; an initial large whisker may split into some whiskers, but does not result into individual laths. After five seconds have passed, all simulations have reached their steady state values. This means that after this time the only thing that happens is a rearrangement of the laths in the whiskers in order to approach the appropriate equilibrium distributions. This process takes about twenty seconds. The difference of the total material in whiskers with respect to the temperature comes from the fact that at higher temperatures there is more chance that there are single laths not belonging to any cluster.

**Fig 10:**
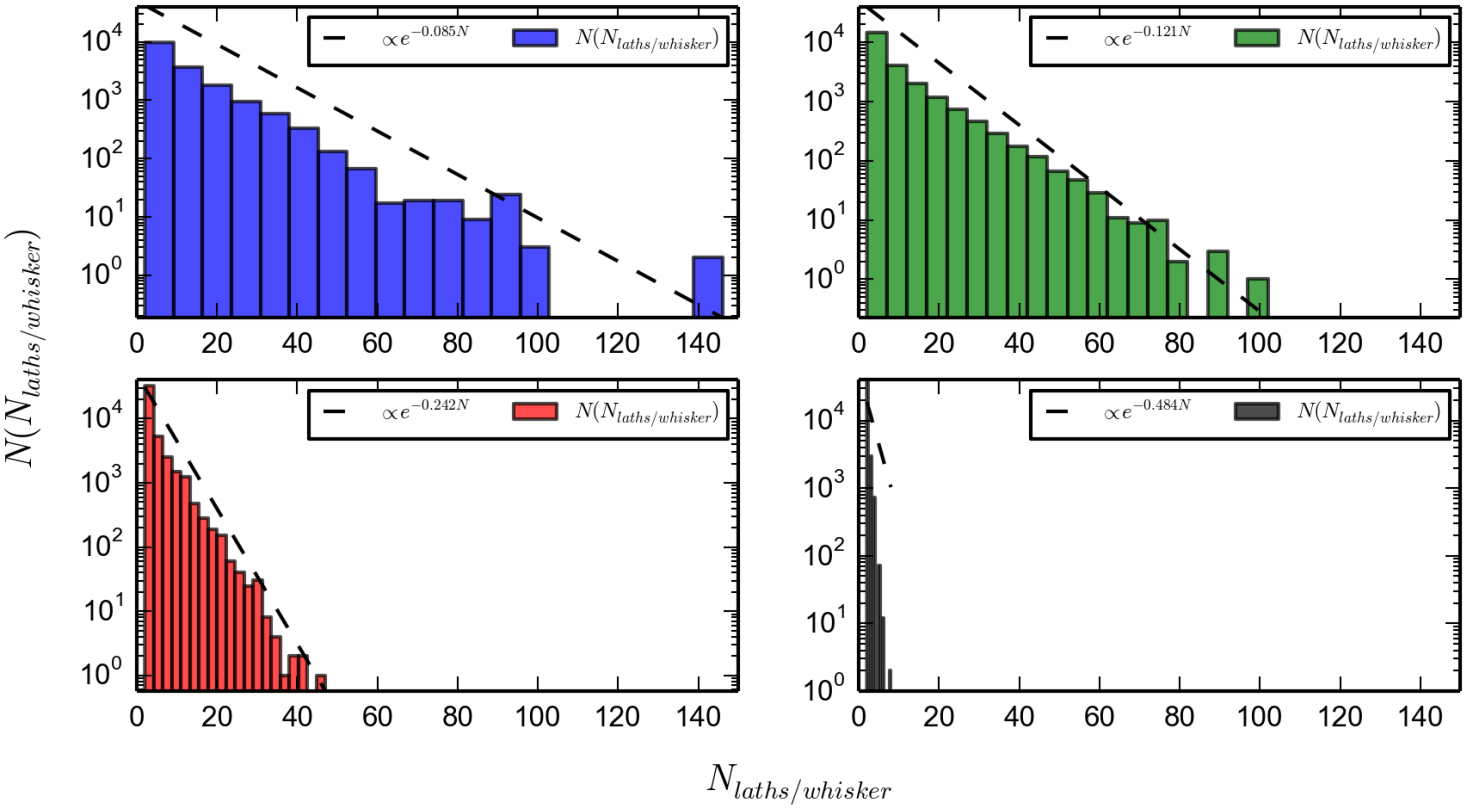
Histogram of laths per whisker. Simulations are equilibrated by running 40 *s*, and continued by 40 *s*. The dashed line represents an exponential function, $e^{-\frac{N}{{\bar{N}}_{laths/whisker}}}$ indicating an exponential distribution with $\lambda =1/{\bar{N}}_{laths/whisker}$. *(Upper left)*, *T* = 0.1 *T*_0_, $\lambda =1/{\bar{N}}_{laths/whisker}=1./11.68=0.8556$, mean ${\bar{N}}_{laths/whisker}=11.68$; *(upper right)*, *T* = 0.3 *T*_0_, *λ* = 1./8.28 = 0.120, mean ${\bar{N}}_{laths/whisker}=8.28$; *(lower left)*, *T* = 0.5 *T*_0_, *λ* = 1./4.13 = 0.2421, mean ${\bar{N}}_{laths/whisker}=4.13$; *(lower right)*, *T* = 1.9 *T*_0_, *λ* = 1./2.064 = 0.4845, mean ${\bar{N}}_{laths/whisker}=2.064$.

**Fig 11:**
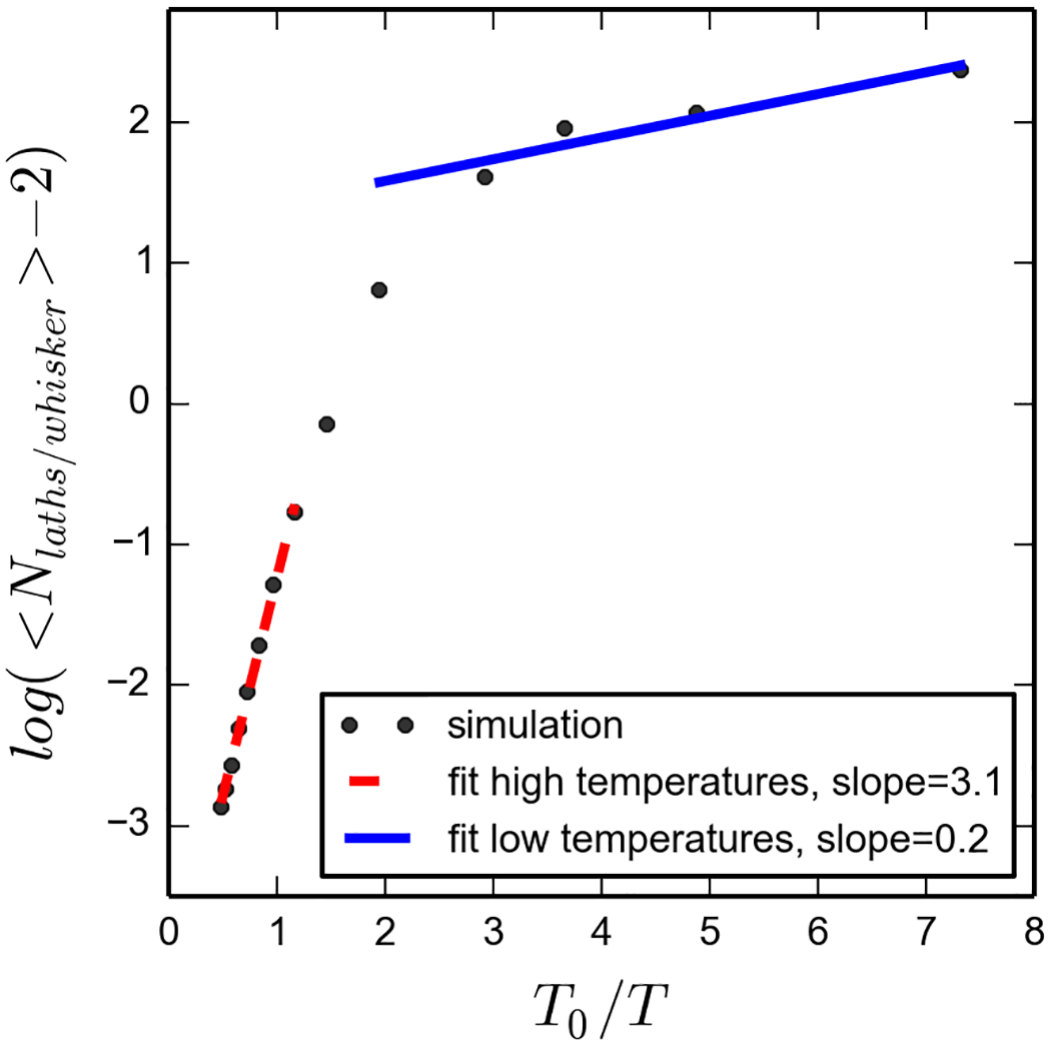
Average number of laths per whisker against *T*_0_/*T*. The simulation was run for 40*s* for equilibration and then continued for another 100 *s*.

**Fig 12:**
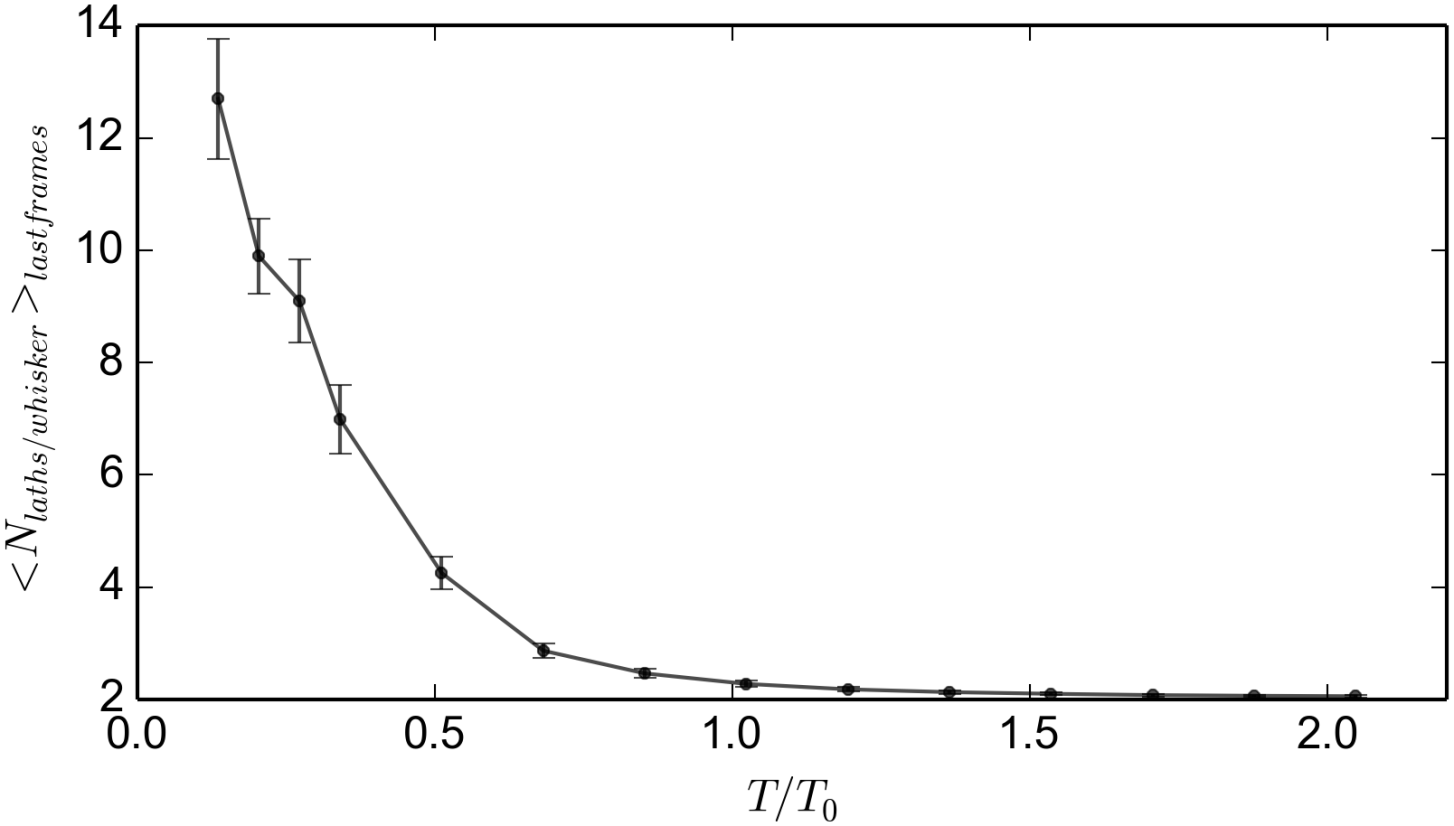
Average number of laths per whisker. The simulation was run for 40*s* for equilibration and then continued for another 100 *s*. The independent variable is *T*/*T*_0_.

Next, we are interested in the length-distributions of the whiskers in equilibrium. In Fig 10, upper right, we present the distribution of lengths in boxes with temperature 0.2*T*_0_. The plot is based on the results of 10 simulations, starting with different seeds for the random number generators and equilibrated for 40 *s*, after which production runs of 40 *s* each were started. Notice how the lengths of whiskers are exponentially distributed, with a mean value of 8.28 laths per whisker for the 0.2*T*_0_ value of the temperature (and similar exponential distribution for other values of the temperature). This implies a slope in Fig 10 of *λ* = 1/8.28 ≈ 0.121. The exponential distribution allows for only very few long whiskers; which at this temperature have lengths of about 90 laths. Nevertheless, such long whiskers quickly deplete the pool of laths, leading to severe finite size effects for temperatures of about 0.2*T*_0_ or less. This is seen in Figs 11 and 12 where we have plotted the average lengths of whiskers as a function of inverse temperature. Notice also that a whisker of length 8, (as, for instance, the orange at the middle of the leftmost box of Fig 4), would not span the simulation box; its length being of the order of 0.4 ∗ 8 ∗ *L*_*lath*_ ≈ 0.219*μm* compared to the 0.542*μm* for the length of the side of the simulation box in Table 1. The fraction of whiskers whose length is enough as to percolate the simulation box, at this temperature, is 0.083; with 20 laths or more per whisker. As the temperature is increased, the medium value of the length of the whiskers is decreased and long whiskers are less common. This can be seen in Fig 10, by comparing the low temperatures (*T* ≈ 0.2, upper left and *T* ≈ 0.4, upper right) with the high temperatures (*T* ≈ 0.5, lower left and *T* ≈ 1.9 lower right).

In BOLD, the asymptotic expressions relating the average number of laths per whisker and the temperature, as shown in the S4 File, are:

$$\begin{array}{ccc} \ln(N_{laths/whisker}-2) & = & \frac{1}{2}\ln\phi +\frac{\epsilon }{2k_{B}T}\;\;\;\text{for}\;\text{low}\;\text{temperatures}\;\\ \ln(N_{laths/whisker}-2) & = & \ln\phi +\frac{\epsilon }{k_{B}T}\;\;\;\text{for}\;\text{high}\;\text{temperatures}\; \end{array}$$ (9)

Where *ϕ* is the volume fraction. The equilibrium distribution of whisker lengths in the present system is analogous to the distribution of length of worm-like micelles studied by Cates and Candau [31]. For low temperatures the equation is the same (compare Eq (9) to Cates and Candau equation (2.4b)). Our system recovers, quantitatively, the expected trend for high temperatures, as seen in Fig 11, that is $\ln<N_{laths/whisker}>\propto \frac{1}{T}$. The constant, though, does not mach; the theoretical value for the slope of the red dashed line being *ϵ*/(*K*_*B*_ *T*_0_) = 100. There is a large discrepancy between our results and the approximated theory (of Eq (9)), of the order of 30. We do not have yet a final explanation, however it may come from the fact that the boxes have very low number of laths in the simulation box compared to the real system; with only 512 of them, so there may be size effects. This effect is also amplified if we take into the fact that appearance of large whiskers is a rare event. Further work at larger systems could help to solve this issue.

Another quantity that characterizes the system is the long-time diffusivity, measured from the mean square displacement. In Table 2 we show the diffusion constant at different temperature values. At low temperatures it is close to zero, and increases with temperature.

**Table 2:** Diffusion constant. The system was run during 40 *s*, other parameters in Table 1.

*T* (*T*_0_)	*D* (*μm*)^2^/*s*
0.1*T*_0_	0.30 ± 0.28
0.3*T*_0_	0.90 ± 0.57
0.5*T*_0_	2.6 ± 1.5
0.8*T*_0_	7.7 ± 2.2
1.2*T*_0_	9.5 ± 2.3
1.9*T*_0_	13.0 ± 6.5

### Rheology and gel transition

In this section we study the rheological properties BOLD. In particular we investigate how the shear relaxation modulus varies with temperature, and how these variations are related to changes of the distribution of whiskers.

**Fig 13:**
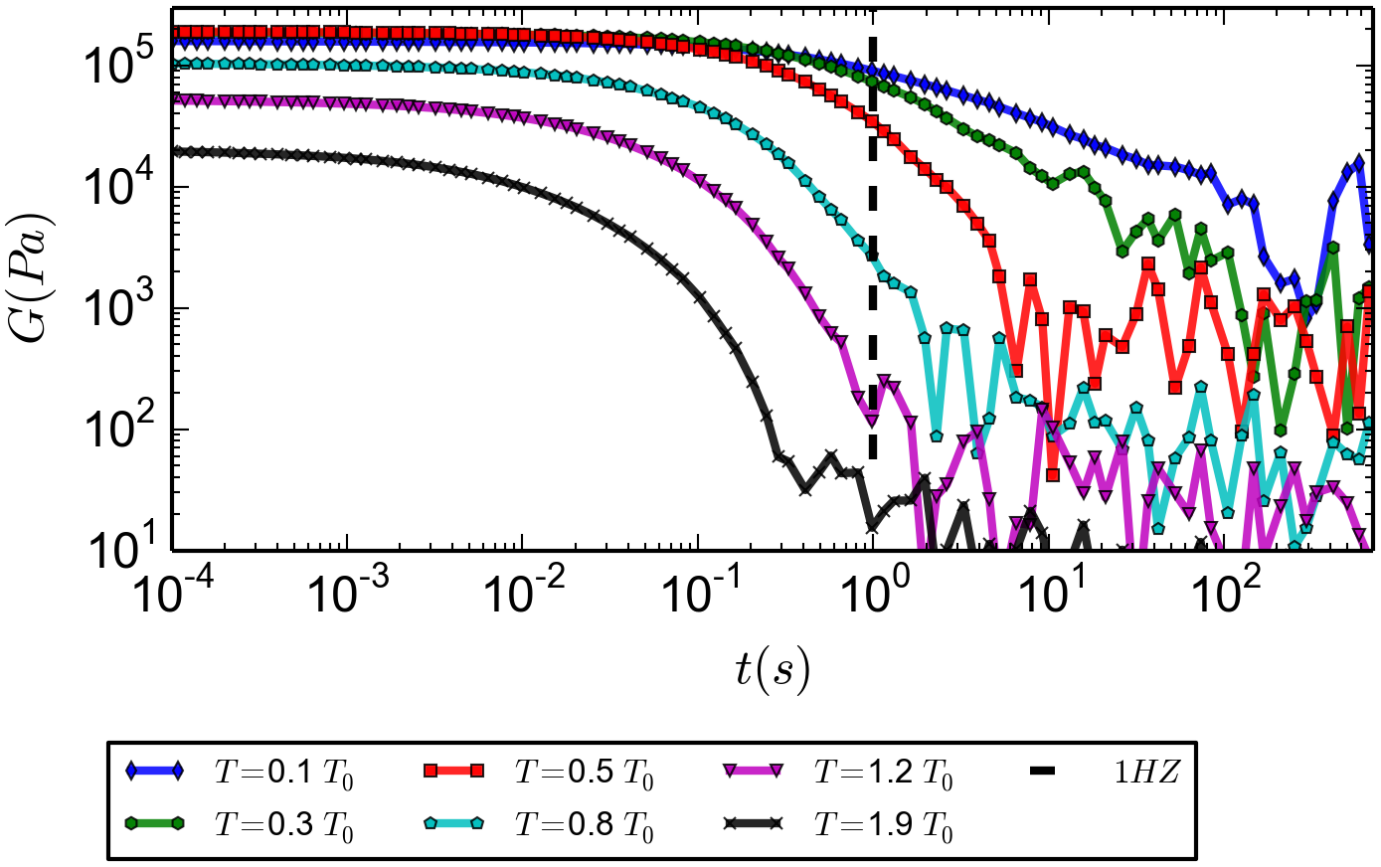
Shear relaxation modulus, *G*(*t*), for different values of the temperature of the system. The system was equilibrated (during 40 *s*); this data was taken after it had run for another 100 *s*. *T* in Kelvin.

At Fig 13 we present the shear relaxation modulus *G*(*t*) as a function of time for various temperatures. Since we are not aiming to study rheological properties like the shear storage and loss modulus, *G*′ and *G*′′ respectively, in great detail, we did not push calculations of the shear relaxation modulus to convergence for times larger than a few seconds. It is clear from Fig 13 that the plateau value at early times quickly increases with decreasing temperature (or at decreasing value of the diffusion constant).

**Fig 14:**
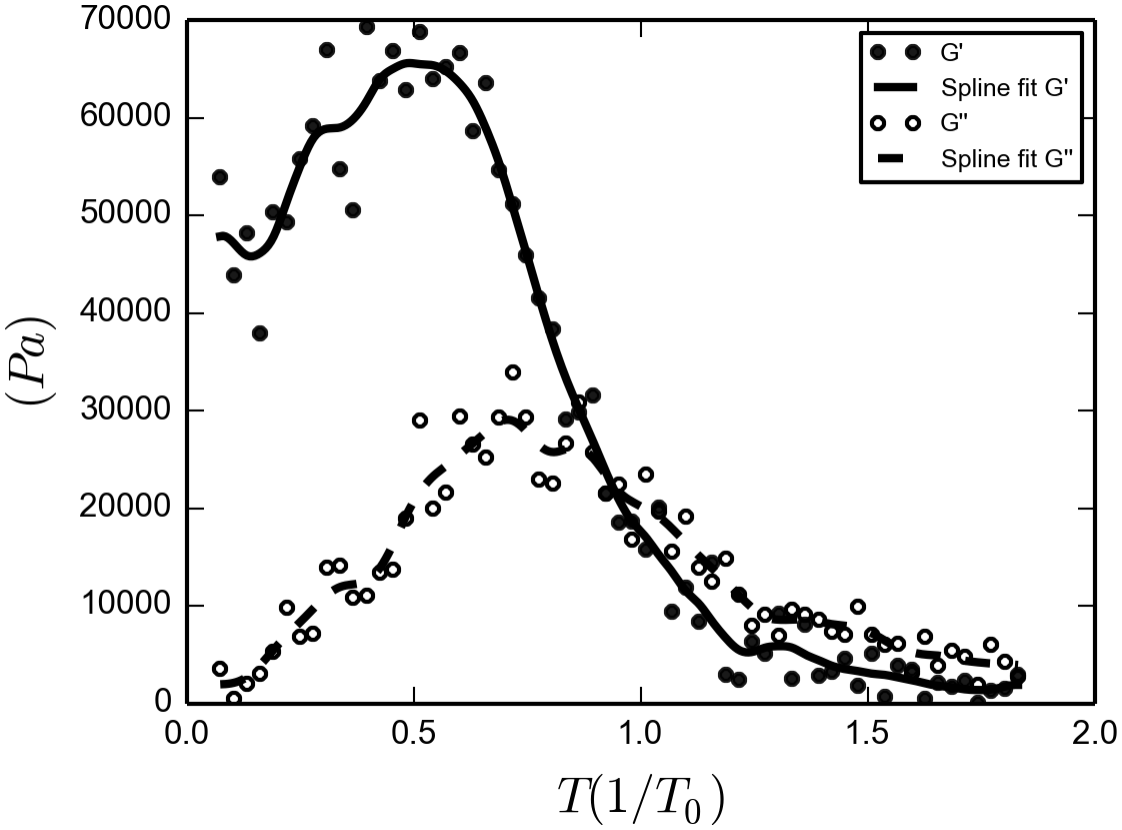
Oscillatory shear numerical experiment. We ran 60 × 10^5^ time steps (60 *s*) discretely increasing the temperature each 1 × 10^5^ steps (1 *s*), in 8 *K* each time, a rate of 8 *K*/*s*.

A possible way to experimentally determine a gel transition is to compare the values of *G*′ and *G*′′ at a frequency of one Hertz, (as discussed in [2], pg. 15 and 16. Furthermore, the experiments of P3HT by Newbloom et. al., [4], were conducted at this frequency). In the plot of the shear relaxation modulus as a function of time (Fig 13), we notice that at times less than about one second high temperature *G*(*t*)′*s* have decayed to very small values, while low temperature *G*(*t*)′*s* have not decayed at all. At intermediate temperatures the shear relaxation modulus for *t* = 1*s* sharply rises. Therefore, in Fig 14 we plot the storage and loss modulus at one Hertz as a function of temperature. An oscillatory shear experiment that measures *G*′ and *G*′′ while slowly decreasing the temperature 8 *K* (about 0.03*T*_0_) each 1 *s*, gives rise to a transition from liquid to gel (imposing a decrease in temperature implies that it is not a strictly closed system, so its entropy does not need to increase). The measurement of *G*′ and *G*′′ was made using the methods presented in the discussion of Eq (8). This numerical experiment can be compared directly with Figure 1. of [4]. It is clear from our plot that for temperatures below about 0.7*T*_0_ the system behaves basically elastic, while for temperatures above 1.1*T*_0_ the system is predominantly viscous. We therefore conclude that according to the usual rheological definition a gel transition occurs at about 0.9*T*_0_ (or 250*K*), even though, there is no branching and barely any percolating whiskers in the system (around 8% at a low temperature of 0.2*T*_0_, as calculated from Fig 10). As a consistency test, this results were tested again at 2Hz, and a similar curve is obtained (Included in the S5 File.)

Given the fact that our model has to be taken as highly coarse grained with relation to polymers, we find it remarkable the fact that this behavior is quite similar to that reported in rheological experiments of gelation of P3HT [4]. Both in the experimental oscillatory rheology and in the numerical experiment there is a temperature dependent gelation. The main drawback of our model with respect to the experiment by Newbloom et. al. is the absence of hysteresis in our model; in other words, our simulations do not behave differently if the path to equilibrium is achieved by heating or by cooling down. This may be caused by the lack of branching of our system. Not having this possibility makes the system pseudo-one dimensional; and since there are no phase transitions for one dimensional structures, while for higher dimensional there are both percolation and phase transitions, it is expected that branching would provide such transitions.

From these results we come to the conclusion that, establishing whether whisker forming systems present a gel transition or not, depends very much on the definition of gel transition being used.

Based on our results, the gel transition of whisker forming systems highly depends on the definition of gel transition itself. From the structural point of view, there is no branching and one can hardly talk of a network of whiskers spanning the whole simulation box, since long whiskers are rare. However, from the rheological point of view, there is a gelation process. Finally, we expect a lower gel transition temperature in a less controlled laboratory setting, given the fact that a real system will be affected by different scales of strengths and different frequencies of oscillation.

## Conclusion

We have investigated the gel transition in stacking long laths, being prototype for moderately long stiff aromatic polymers. The stacking bonds allow for the creation of long whiskers of consecutively stacked laths. The whisker formation process that reaches an equilibrium state in matter of 20*s* to 25*s*, depending on the temperature. We do not allow for branching of such whiskers, thereby preventing the occurrence of extended three-dimensional networks. The pseudo one-dimensional character of the whiskers does not allow for structural transitions with varying temperatures that could depend on the direction of the temperature gradient; in other words, hysteresis is not present in the gel-sol transition. The distribution of number of laths per whisker, which depends on the temperature, has an exponential shape. For *T* = 0.3*T*_0_, this gives a mean of 8.28 laths per whisker; similar to the case of worm-like micelles. As a result, only very few long whiskers occur amidst a sea of many smaller whiskers. Chances of having mechanically percolating structures are therefore very low. Nevertheless, we have found that with the usual definition of gel transition, occurring at a temperature where the ratio of the storage and loss modulus at a frequency of 1*Hz* changes from values larger than one to values lower than one, such a transition indeed does occur. Our main conclusion therefore is that the occurrence of a gel transition does not necessarily imply that percolating three dimensional structures have developed in the system. Further improvements to the present model include the study of the interaction between sphere-like particles, like the PCBM or C60, with the laths. In this case the possibility of branching also should be taken into account. It could also be important to study the effect on the alignment that can be obtained by including a second kind of lath, that not necessarily shares the aromatic interaction; as for instance gold nanorods [32].

## Supporting information

S1 File Appendix: Forces. Includes the expressions for the forces.
(PDF)

S2 File Appendix: Torques. Includes the expressions for the torques.
(PDF)

S3 File Appendix: *π* − *π* interaction energy. Includes the discussion of the value of the interaction energy.
(PDF)

S4 File Appendix: Asymptotic expressions for number of laths per whisker. Analytic calculation of asymptotic value of number of laths per whisker.
(PDF)

S5 File Appendix: Supplementary results. As consistency test, we measured storage and loss modulus at two Hertz.
(PDF)
