# Supplementary material for: Brownian orientational lath model (BOLD): A computational model relating the self-assembly in a fluid of lath like particles with its rheology and gelation
Source: PLoS One. 2018 Feb 7;13(2):e0191785. doi: 10.1371/journal.pone.0191785 (PMC5802906; doi:10.1371/journal.pone.0191785)
Supplement: S1 File — Includes the expressions for the forces. (PDF) [file pone.0191785.s001.pdf]

Supporting Information for Brownian orientational lath model (BOLD): a computational model relating the self-assembly in a fluid of lath like particles with its rheology and gelation. Appendix S1: Forces

Gabriel Villalobos[1,2*]

1 Computational Biophysics, University of Twente, P.O. Box 217, 7500 AE, Enschede, The Netherlands
2 Universidad de Bogotá Jorge Tadeo Lozano, Departamento de Ciencias Básicas. Carrera 4 Número 22 - 61. Módulo 6, oficina 501. 110311. Bogotá, Colombia.

* gabriel.villalobosc@utadeo.edu.co

Supporting information

Appendix S1: forces The force acting on lath i is:

$$\mathbf{F} = -\frac{\partial \Phi_S}{\partial \mathbf{R}_i}$$

Since the potential is the product of different functions is useful to go over their derivatives with respect to R_i. Take first V_d:

$$-\frac{\partial V_d(r_{kj})}{\partial \mathbf{R}_i} = \frac{\epsilon}{2}\left(\frac{-a}{\cosh^2(a(r_{kj}-\frac{\sigma}{2}))\tanh\left(\frac{a\sigma}{2}\right)} \times \frac{\mathbf{r}_{jk}}{|\mathbf{r}_{jk}|}(\delta_{ik}-\delta_{ij})\right) \tag{1}$$

The V_{cn} potential takes two forms, depending on the $\hat{\mathbf{n}}_k$ vector, either :

$$(\hat{\mathbf{n}}_{k,j,k}\cdot\hat{\mathbf{r}}_{k,j})^{2l} \tag{2}$$

or

$$(\hat{\mathbf{n}}_{s(k),k,k}\cdot\hat{\mathbf{r}}_{k,j})^{2l} \tag{3}$$

One way to calculate the stress components during the simulation, taking into account the periodic boundary conditions, requires to explicitly write the forces as derivatives with respect to the relative vectors $\mathbf{r}_{kj}$ and $\mathbf{r}_{s(k),k}$. As Eq (2) depends on $\mathbf{r}_{kj}$ (there is no $s(k)$ lath to take into account here), then the derivative with respect to either k or j is:

$$\begin{aligned}
-\frac{\partial}{\partial \mathbf{R}_i}\left(\frac{\mathbf{n}_{k,j,k}\cdot\mathbf{r}_{k,j}}{|\mathbf{n}_{k,j,k}||\mathbf{r}_{k,j}|}\right)^{2l} &= \Bigg[\frac{4l(\mathbf{n}_{k,j,k}\cdot\mathbf{r}_{k,j})^{2l-1}}{(|\mathbf{n}_{k,j,k}||\mathbf{r}_{k,j}|)^{2l}}\mathbf{n}_{k,j,k} \\
&- \frac{2l(\mathbf{n}_{k,j,k}\cdot\mathbf{r}_{k,j})^{2l}}{(|\mathbf{n}_{k,j,k}||\mathbf{r}_{k,j}|)^{2l+1}}\left(|\mathbf{r}_{k,j}|\hat{\mathbf{n}}_{k,j,k}+|\mathbf{n}_{k,j,k}|\hat{\mathbf{r}}_{k,j}\right)\Bigg] \\
&\times \quad (\delta_{i,k}-\delta_{i,j})
\end{aligned} \tag{4}$$

In the case that the potential is Eq (3), the derivative with respect to the coordinates of lath k is:

$$\frac{\partial}{\partial \mathbf{R}_i}\left(\frac{\mathbf{n}_{s(k),k,k}\cdot\mathbf{r}_{k,j}}{|\mathbf{n}_{k,j,k}||\mathbf{r}_{k,j}|}\right)^{2l} = \frac{\partial}{\partial \mathbf{r}_{kj}}\left(\frac{\mathbf{n}_{s(k),k,k}\cdot\mathbf{r}_{k,j}}{|\mathbf{n}_{k,j,k}||\mathbf{r}_{k,j}|}\right)^{2l}\frac{\partial \mathbf{r}_{kj}}{\partial \mathbf{R}_k} + \frac{\partial}{\partial \mathbf{r}_{k}}\left(\frac{\mathbf{n}_{s(k),k,k}\cdot\mathbf{r}_{k,j}}{|\mathbf{n}_{k,j,k}||\mathbf{r}_{k,j}|}\right)^{2l}\frac{\partial \mathbf{r}_{s(k),k}}{\partial \mathbf{R}_k} \tag{5}$$

Obtaining:

$$\frac{\partial}{\partial \mathbf{r}_{kj}}\left(\frac{\mathbf{n}_{s(k),k,k}\cdot\mathbf{r}_{k,j}}{|n_{k,j,k}||r_{k,j}|}\right)^{2l} = \frac{2l[\mathbf{n}_{s(k),k,k}\cdot\mathbf{r}_{k,j}]^{2l-1}}{[|\mathbf{r}_{k,j}||\mathbf{n}_{s(k),k,k}|]^{2l}}\mathbf{n}_{s(k),k,k} - \frac{2l[\mathbf{n}_{s(k),k,k}\cdot\mathbf{r}_{kj}]^{2l}}{[|\mathbf{r}_{k,j}||\mathbf{n}_{s(k),k,k}|]^{2l+1}}|\mathbf{n}_{s(k),k,k}|\hat{\mathbf{r}}_{k,j} \tag{6}$$

$$\frac{\partial}{\partial \mathbf{r}_{s(k),k}}\left(\frac{\mathbf{n}_{s(k),k,k}\cdot\mathbf{r}_{k,j}}{|n_{k,j,k}||r_{k,j}|}\right)^{2l} = \frac{2l[\mathbf{n}_{s(k),k,k}\cdot\mathbf{r}_{k,j}]^{2l-1}}{[|\mathbf{r}_{k,j}||\mathbf{n}_{s(k),k,k}|]^{2l}}\mathbf{n}_{k,j,k} - \frac{2l[\mathbf{n}_{s(k),k,k}\cdot\mathbf{r}_{kj}]^{2l}}{[|\mathbf{r}_{k,j}||\mathbf{n}_{s(k),k,k}|]^{2l+1}}|\mathbf{r}_{k,j}|\hat{\mathbf{n}}_{s(k),k,k} \tag{7}$$

and :$\frac{\partial \mathbf{r}_{s(k),k}}{\partial \mathbf{R}_k} = 1$, $\frac{\partial \mathbf{r}_{kj}}{\partial \mathbf{R}_k} = -1$. The derivative with respect to j,k or $s(k)$ is:

$$-\frac{\partial}{\partial \mathbf{R}_i}\left(\frac{\mathbf{n}_{s(k),k,k}\cdot\mathbf{r}_{k,j}}{|n_{k,j,k}||r_{k,j}|}\right)^{2l} = \frac{2l[\mathbf{n}_{s(k),k,k}\cdot\mathbf{r}_{k,j}]^{2l-1}}{[|\mathbf{r}_{k,j}||\mathbf{n}_{s(k),k,k}|]^{2l}}\big(\mathbf{n}_{j,k,k}(\delta_{i,k}-\delta_{i,s(k)}) + \mathbf{n}_{s(k),k,k}(\delta_{i,k}-\delta_{i,j})\big) + \frac{2l[\mathbf{n}_{s(k),k,k}\cdot\mathbf{r}_{kj}]^{2l}}{[|\mathbf{r}_{k,j}||\mathbf{n}_{s(k),k,k}|]^{2l+1}}\big(|\mathbf{r}_{k,j}|\hat{\mathbf{n}}_{s(k),k,k}(\delta_{i,k}-\delta_{i,s(k)}) - |\mathbf{n}_{s(k),k,k}|\hat{\mathbf{r}}_{k,j}(\delta_{i,k}-\delta_{i,j})\big) \tag{8}$$
