# Supplementary material for: Brownian orientational lath model (BOLD): A computational model relating the self-assembly in a fluid of lath like particles with its rheology and gelation
Source: PLoS One. 2018 Feb 7;13(2):e0191785. doi: 10.1371/journal.pone.0191785 (PMC5802906; doi:10.1371/journal.pone.0191785)
Supplement: S2 File — Includes the expressions for the torques. (PDF) [file pone.0191785.s002.pdf]

Supporting Information for Brownian orientational lath model (BOLD): a computational model relating the self-assembly in a fluid of lath like particles with its rheology and gelation. Appendix S2: Torques

Gabriel Villalobos[1,2*]

1 Computational Biophysics, University of Twente, P.O. Box 217, 7500 AE, Enschede, The Netherlands
2 Universidad de Bogotá Jorge Tadeo Lozano, Departamento de Ciencias Básicas. Carrera 4 Número 22 - 61. Módulo 6, oficina 501. 110311. Bogotá, Colombia.

* gabriel.villalobosc@utadeo.edu.co

Supporting information

Appendix S2: torques The definition of the torque is:

$$\mathbf{T}_{i,stk.} = -\hat{\mathbf{u}}_i \times \frac{\partial \Phi_S}{\partial \hat{\mathbf{u}}_i}$$

And given the torque, the evolution of the orientation vector is given by:

$$d\hat{\mathbf{u}} = \underbrace{[\hat{I} - \hat{\mathbf{u}}\hat{\mathbf{u}}] \cdot \Gamma \cdot \hat{\mathbf{u}}dt}_{\delta u_{i_1}} + \underbrace{\frac{1}{\gamma}\mathbf{T} \times \hat{\mathbf{u}}dt}_{\delta u_{i_2}} \tag{1}$$

With δ_{u_1} related to the flow. Both V_o and V_{cn} depend on the orientation $\hat{\mathbf{u}}$.

$$\begin{aligned} -\left(\hat{\mathbf{u}}_i \times \frac{\partial V_{so}(\mathbf{r}_{k,j}, \mathbf{r}_{s(k),k})}{\partial \hat{\mathbf{u}}_i}\right) \times \hat{\mathbf{u}}_i &= 2l\delta_{i,k}\left(\frac{[\mathbf{n}_{s(k),k,k} \cdot \mathbf{r}_{kj}]^{2l-1}}{[|\mathbf{n}_{s(k),k,k}||\mathbf{r}_{k,j}|]^{2l}}\left(\mathbf{n}_{s(k),k,k}(\mathbf{r}_{k,j} \cdot \hat{\mathbf{u}}_k)\right.\right. \\ &+ \left.\mathbf{n}_{k,j,k}(\hat{\mathbf{u}}_k \cdot \mathbf{r}_{s(k),k})\right) \\ &- \left.\frac{[\mathbf{n}_{s(k),k,k} \cdot \mathbf{r}_{kj}]^{2l}}{|\mathbf{n}_{s(k),k,k}|^{2l+2}|\mathbf{r}_{k,j}|^{2l}}\mathbf{n}_{s(k),k,k}(\mathbf{r}_{s(k),k} \cdot \hat{\mathbf{u}}_k)\right) \end{aligned} \tag{2}$$

For the case in which the orientation vector $\mathbf{n}_k$ is not given by a stuck lath the potential is $V_{cn}(\hat{\mathbf{n}}_{k,j,k} \cdot \hat{\mathbf{r}}_{k,j})$, and the analogous calculation gives:

$$\begin{aligned} - & \left(\hat{\mathbf{u}}_i \times \frac{\partial V_{so}(\mathbf{r}_{k,j}, \mathbf{r}_{s(k),k})}{\partial \hat{\mathbf{u}}_i}\right) \times \hat{\mathbf{u}}_i = \\ 2l & \delta_{i,k}\left(\frac{(\mathbf{n}_{k,j,k} \cdot \mathbf{r}_{k,j})^{2l-1}}{[|\mathbf{n}_{k,j,k}||\mathbf{r}_{k,j}|]^{2l}} 2\mathbf{n}_{k,j,k}(\mathbf{r}_{k,j} \cdot \hat{\mathbf{u}}_k) - \frac{(\mathbf{n}_{k,j,k} \cdot \mathbf{r}_{k,j})^{2l}}{|\mathbf{n}_{k,j,k}|^{2l+2}|\mathbf{r}_{k,j}|^{2l}}\mathbf{n}_{k,j,k}(\mathbf{r}_{k,j} \cdot \hat{\mathbf{u}}_k)\right) \end{aligned} \tag{3}$$
