# Supplementary material for: Brownian orientational lath model (BOLD): A computational model relating the self-assembly in a fluid of lath like particles with its rheology and gelation
Source: PLoS One. 2018 Feb 7;13(2):e0191785. doi: 10.1371/journal.pone.0191785 (PMC5802906; doi:10.1371/journal.pone.0191785)
Supplement: S3 File — Includes the discussion of the value of the interaction energy. (PDF) [file pone.0191785.s003.pdf]

Supporting Information for Brownian orientational lath model (BOLD): a computational model relating the self-assembly in a fluid of lath like particles with its rheology and gelation. Appendix S3: $\pi - \pi$ interaction energy

Gabriel Villalobos[1,2*]

1 Computational Biophysics, University of Twente, P.O. Box 217, 7500 AE, Enschede, The Netherlands
2 Universidad de Bogotá Jorge Tadeo Lozano, Departamento de Ciencias Básicas. Carrera 4 Número 22 - 61. Módulo 6, oficina 501. 110311. Bogotá, Colombia.

* gabriel.villalobosc@utadeo.edu.co

Supporting information

Appendix S3: $\pi - \pi$ interaction energy Using MP2, Tsuzuki et al. [1], found an energy of interaction of the thiophene dimer of -1.71 $kcal/mol$ for the parallel plate geometry. Using also MP2, [2] reports -3 $kcal/mol$. We should also mention the work by Dag et. al., [3], who reported an energy of interaction of -60.57 kJ/mol (about 14.47 $kcal/mol$), using DFT generalized gradient approximation. Notwithstanding, Rodríguez-Ropero et al. performed calculations using MP2, MP3, MP4 and CCSD(T). They compare the interaction energy, and claim that there is agreement between MP4 and CCSD(T), while MP2 "significantly overestimates the ΔE_{Int} compared to CCSD(T)" [4]. In the present paper we have used their value for the interaction which is -0.34 $kcal/mol$.

References

1. Tsuzuki S, Honda K, Azumi R. Model Chemistry Calculations of Thiophene Dimer Interactions: Origin of π-Stacking. JAmChemSoc. 2002;124(41):12200 – 12209.

2. Scherlis DA, Marzari N. π-Stacking in Thiophene Oligomers as the Driving Force for Electroactive Materials and Devices. Journal of the American Chemical Society. 2005;127(9):3207–3212. doi:10.1021/ja043557d.

3. Dag S, Wang LW. Packing Structure of Poly(3-hexylthiophene) Crystal: Ab Initio and Molecular Dynamics Studies. The Journal of Physical Chemistry B. 2010;114(18):5997–6000. doi:10.1021/jp1008219.

4. Rodríguez-Ropero F, Casanovas J, Alemán C. Ab initio calculations on π-stacked thiophene dimer, trimer, and tetramer: Structure, interaction energy, cooperative effects, and intermolecular electronic parameters. Journal of Computational Chemistry. 2008;29(1):69–78. doi:10.1002/jcc.20763.
