# Supplementary material for: Brownian orientational lath model (BOLD): A computational model relating the self-assembly in a fluid of lath like particles with its rheology and gelation
Source: PLoS One. 2018 Feb 7;13(2):e0191785. doi: 10.1371/journal.pone.0191785 (PMC5802906; doi:10.1371/journal.pone.0191785)
Supplement: S4 File — Analytic calculation of asymptotic value of number of laths per whisker. (PDF) [file pone.0191785.s004.pdf]

Supporting Information for Brownian orientational lath model (BOLD): a computational model relating the self-assembly in a fluid of lath like particles with its rheology and gelation. Appendix S4: Asymptotic expressions for number of laths per whisker

Gabriel Villalobos[1,2*]

1 Computational Biophysics, University of Twente, P.O. Box 217, 7500 AE, Enschede, The Netherlands
2 Universidad de Bogotá Jorge Tadeo Lozano, Departamento de Ciencias Básicas. Carrera 4 Número 22 - 61. Módulo 6, oficina 501. 110311. Bogotá, Colombia.

* gabriel.villalobosc@utadeo.edu.co

Supporting information

Appendix S4: Asymptotic expressions for number of laths per whisker
The Free energy per volume of the system reads:

$$A = -K_B T \sum_{n=1}^{\infty} c_n \ln c_n - c_n + \beta\epsilon,$$

with $\beta = \frac{1}{k_B T}$. From now on, $E = \beta\epsilon$. The total number of laths per volume:

$$\phi = \sum_{n=1}^{\infty} n c_n.$$

Then:

$$c_1 = \phi - \sum_{n=2}^{\infty} n c_n.$$

Minimizing the free energy with respect to c_n, with $n = 2, 3, ...$:

$$\{\ln c_n + E\} - n\{\ln c_1 + E\} = 0,$$

or

$$c_n e^E = (c_1 e^E)^n.$$

Defining $x = c_1 e^E$, the total number of laths is given by:

$$\phi e^E = \sum_{n=1}^{\infty} n x^n = \frac{x}{(1-x)^2}.$$

Putting $y = \phi e^E$, solve for x:

$$x = 1 + \frac{1}{2y} \pm \sqrt{\frac{1}{y} + \frac{1}{4y^2}} \approx 1 - \sqrt{\frac{1}{y}}.$$

Were E and therefore y are assumed to be large. Then:

$$c_n = e^{-E} x^n = e^{-E} \left(1 - \sqrt{\frac{1}{y}}\right)^n \approx e^{-E} e^{-n/\sqrt{y}},$$

or:

$$c_n = \frac{\phi}{y} e^{-n/\sqrt{y}}$$

The average number of laths:

$$n_{av} = \frac{\sum_{n=1}^{\infty} n c_n}{\sum_{n=1}^{\infty} c_n} = \frac{\phi}{\frac{\phi}{y} \frac{e^{-1/\sqrt{y}}}{1-e^{-1/\sqrt{y}}}}.$$

Approximately

$$n_{av} \approx y \left(1 - e^{-1/\sqrt{y}}\right) \approx \sqrt{y}$$

In BOLD the whiskers start at length 2, then:

$$< n >= \frac{\sum_{n=2}^{\infty} n c_n}{\sum_{n=2}^{\infty} c_n} = \frac{\phi - c_1}{\frac{\phi}{y} \frac{e^{-1/\sqrt{y}}}{1-e^{-1/\sqrt{y}}} - c_1}.$$

For low temperatures:

$$< n >= n_{av}$$

For high temperatures:

$$< n >= 2 + y$$

From which Eq (9) of the manuscript directly follows.
