# Supplementary material for: Brownian orientational lath model (BOLD): A computational model relating the self-assembly in a fluid of lath like particles with its rheology and gelation
Source: PLoS One. 2018 Feb 7;13(2):e0191785. doi: 10.1371/journal.pone.0191785 (PMC5802906; doi:10.1371/journal.pone.0191785)
Supplement: S5 File — As consistency test, we measured storage and loss modulus at two Hertz. (PDF) [file pone.0191785.s005.pdf]

# Supporting Information for Brownian orientational lath model (BOLD): a computational model relating the self-assembly in a fluid of lath like particles with its rheology and gelation. Appendix S5: Supplementary results

Gabriel Villalobos[1,2*]

**1** Computational Biophysics, University of Twente, P.O. Box 217, 7500 AE, Enschede, The Netherlands
**2** Universidad de Bogotá Jorge Tadeo Lozano, Departamento de Ciencias Básicas. Carrera 4 Número 22 - 61. Módulo 6, oficina 501. 110311. Bogotá, Colombia.

* gabriel.villalobosc@utadeo.edu.co

## Supporting information

**Appendix S5: Supplementary results** As consistency test, we measured storage and loss modulus at two Hertz, the results, which are similar to the experiment at one Hertz, are shown in the S5.1 Fig.

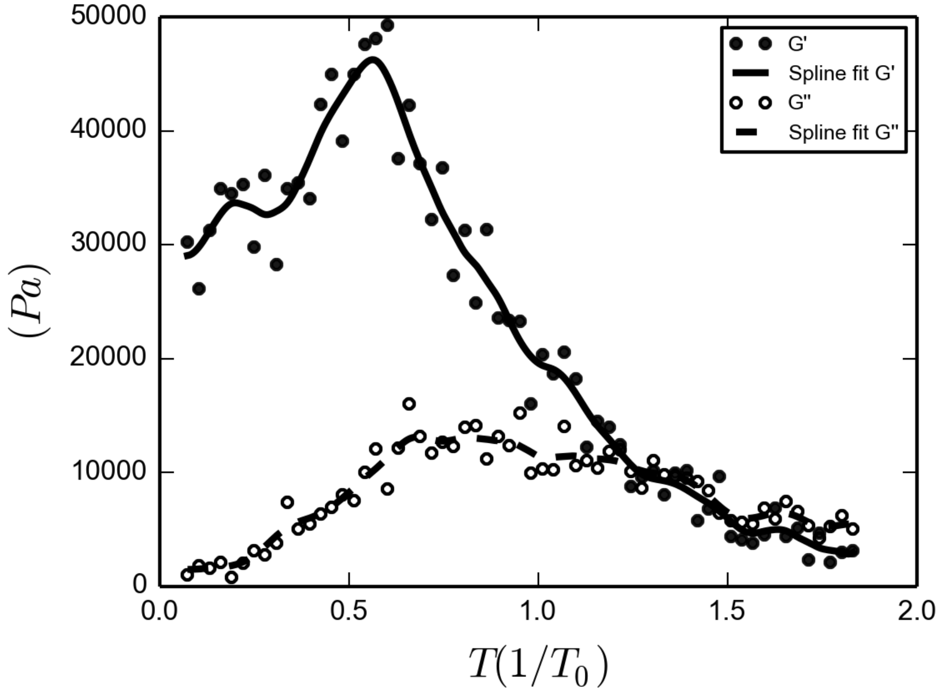


**S5.1 Fig. Oscillatory shear numerical experiment** We have $60 \times 10^5$ time steps ($60\ s$) discretely increasing the temperature each $1 \times 10^5$ steps ($1\ s$), in $8\ K$ each time, a rate of $8\ K/s$, but instead of one Hertz plot the system at two. For this case the rheology is similar than our original system.

The rheology, as measured by the oscillatory shear experiment, does not change strongly with the use of different system sizes. This can be concluded from S5.2 Fig., in which we have used a larger simulation box (Length size $22L_{box}$), but smaller concentration ($c = 0.2$). This implies that the reason of our results is not a strong interaction between the whiskers and their periodic images (since lower concentration would then have an impact on such interactions).

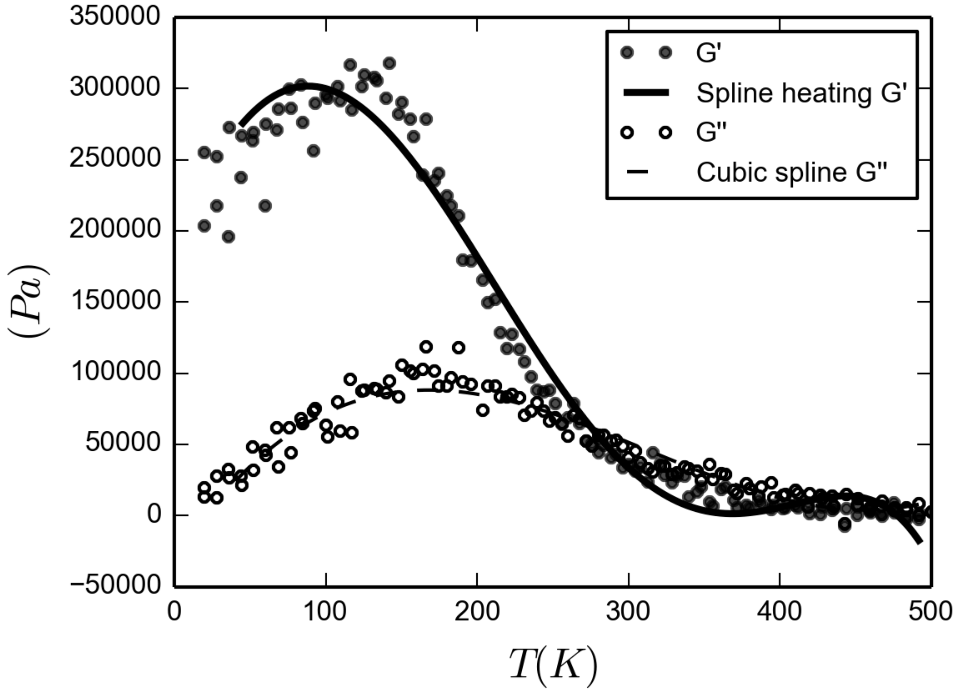


**S5.2 Fig. Oscillatory shear numerical experiment** Here we have $60 \times 10^5$ time steps (60 $s$) discretely increasing the temperature each $1 \times 10^5$ steps (1 $s$), in 8 $K$ each time, a rate of 8 $K/s$. The simulation box length dimension is 22 $L_{lath} = 68.44\mu m$, and the concentration is $c = 0.2$; resulting in 2129 laths. For this larger, more numerous but less concentrated system, the rheology is similar than our original system.

In S5.3 Fig. we present the rheological data from Fig. 14 with the length of the longest whisker as the independent variable. For low vales of the longest whisker the values of G' and G" are similar. As the long whisker length is increased both the storage and loss modulus are increased, up to a certain point in which G' seems to plateau, while G" is reduced. The results are in agreement with Fig. 14, given that as temperature decreases the length of the longer whisker is reduced.

As pointed out by an anonymous reviewer, a possible estimation of the relaxation time is the an order of magnitude calculation for the time required for a particle to explore the simulation box: $(L/2)^2/D_0$. We have calculated this time for 3 different values of the size of the simulation box, and the results are summarized in S[4] Table. For a box whose size is $8L_{lath}$, this time is in agreement with the results of Fig. 6 and Fig. 8. For a smaller system whose length is $6L_{lath}$, the order of magnitude would give a time of about 10 $s$, but longer times are needed to reach equilibrium, as is seen on S5.5 Fig. We conclude that for smaller system size the shape of the decay is similar to the larger system, but the average value of laths per whisker and other quantities are smaller; this latter being an indication of size effects.

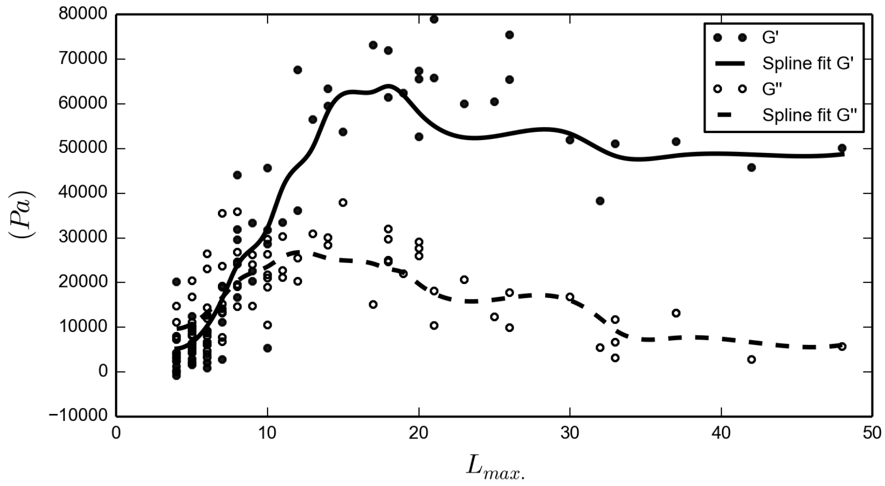


**S5.3 Fig. Oscillatory shear numerical experiment** The rheological data from Fig. 14 is plotted against the length of the longest whisker $L_{max}$.

**S5.4 Table.** Order of magnitude calculation of the relaxation time.

| Length of simulation box | $(L/2)^2/D_0$ |
|---|---|
| $6L_{lath} = 41.1\mu m$ | 9.7 $s$ |
| $8L_{lath} = 54.7\mu m$ | 17.2 $s$ |
| $10L_{lath} = 68.4\mu m$ | 26.9 $s$ |

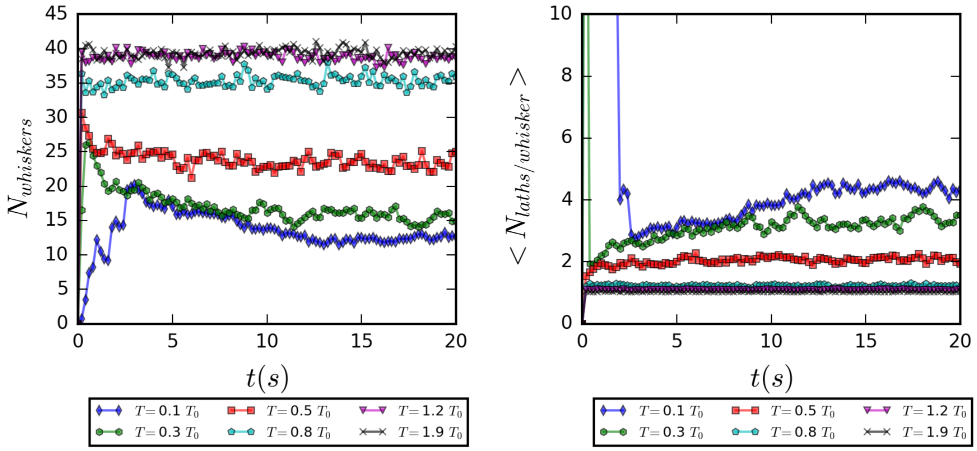


**S5.5 Fig.** Small system of box length $6L_{lath}$. *(Left)* **Number of whiskers as function of time** for different temperatures; average over 10 simulations; initially ordered configuration; ran through 20 $s$. *(Right)* **Number of average laths per whisker** for the same system. (*Color online.*)
